# Human Gingiva-Derived Mesenchymal Stem Cells Modulate Monocytes/Macrophages and Alleviate Atherosclerosis

**DOI:** 10.3389/fimmu.2018.00878

**Published:** 2018-04-30

**Authors:** Ximei Zhang, Feng Huang, Weixuan Li, Jun-long Dang, Jia Yuan, Julie Wang, Dong-Lan Zeng, Can-Xing Sun, Yan-Ying Liu, Qian Ao, Hongmei Tan, Wenru Su, Xiaoxian Qian, Nancy Olsen, Song Guo Zheng

**Affiliations:** ^1^Center for Clinic Immunology, Third Affiliated Hospital at Sun Yat-sen University, Guangzhou, China; ^2^Division of Cardiology, Third Affiliated Hospital at Sun Yat-sen University, Guangzhou, China; ^3^Department of Pathophysiology, Zhongshan School of Medicine, Sun Yat-sen University, Guangzhou, China; ^4^Division of Stomatology, Third Affiliated Hospital at Sun Yat-sen University, Guangzhou, China; ^5^Division of Rheumatology, Penn State Milton S. Hershey Medical Center, Hershey, PA, United States; ^6^Division of Rheumatology, Peking University People’s Hospital, Beijing, China; ^7^Department of Regeneration, Chinese Medical University, Shenyang, China; ^8^Zhongshan Ophthalmic Center, Sun Yat-sen University, Guangzhou, China

**Keywords:** human gingiva-derived mesenchymal stem cells, Ly-6C^hi^ monocytes, macrophages, foam cell, differentiation, atherosclerosis

## Abstract

Atherosclerosis is the major cause of cardiovascular diseases. Current evidences indicate that inflammation is involved in the pathogenesis of atherosclerosis. Human gingiva-derived mesenchymal stem cells (GMSC) have shown anti-inflammatory and immunomodulatory effects on autoimmune and inflammatory diseases. However, the function of GMSC in controlling atherosclerosis is far from clear. The present study is aimed to elucidate the role of GMSC in atherosclerosis, examining the inhibition of GMSC on macrophage foam cell formation, and further determining whether GMSC could affect the polarization and activation of macrophages under different conditions. The results show that infusion of GMSC to AopE^−/−^ mice significantly reduced the frequency of inflammatory monocytes/macrophages and decreased the plaque size and lipid deposition. Additionally, GMSC treatment markedly inhibited macrophage foam cell formation and reduced inflammatory macrophage activation, converting inflammatory macrophages to anti-inflammatory macrophages *in vitro*. Thus, our study has revealed a significant role of GMSC on modulating inflammatory monocytes/macrophages and alleviating atherosclerosis.

## Introduction

Atherosclerosis is a chronic inflammatory disease of the arterial intima characterized by accumulation of lipid and immune cells within the vascular wall (1, 2). Among these immune cells, monocytes/macrophages are the major inflammatory cells involved in modulating atherosclerotic-related inflammation *via* secreting inflammatory cytokines (3–5). Some of the infiltrated monocytes/macrophages take up modified low-density lipoprotein (LDL) particles, such as oxidized LDL (ox-LDL), and further transform into foam cells, which are recognized as the early pathological change of atherosclerosis (6, 7). During foam cell formation, cholesterol uptake mediated by scavenger receptors, such as CD36 and scavenger receptor A (SRA), and cholesterol efflux mediated by ATP-binding cassette transporter A1 (ABCA1) are critical to maintain lipid homeostasis in macrophages (8, 9). Foam cells are formed and they bring the onset of atherosclerosis only when this balance is disturbed. Thus, modulating these factors may help to improve the prevention and treatment of atherosclerosis (10, 11).

It is widely accepted that circulating Ly-6C^hi^ monocytes are precursors of inflammatory macrophages and key participants in chronic inflammation (12, 13). In atherosclerosis, lesion macrophages are also primarily derived from circulating Ly-6C^hi^ monocytes (14–17). More than 90% of monocytes accumulating in atherosclerotic lesions originate from the Ly-6C^hi^ subset instead of the Ly-6C^lo^ subset (18). Upon lesion infiltration, Ly-6C^hi^ monocytes differentiated into lesion macrophages and secreted inflammatory cytokines. Eventually, they may ingest lipids and become foam cells (19). CCR2, the monocyte receptor for monocyte chemoattractant protein-1, mediated the directed migration of Ly-6C^hi^ monocytes into atherosclerotic arteries (20). The chemokine receptor CX3CR1 is also able to mediate direct adhesion of Ly-6C^hi^ monocytes to or migrate toward soluble CX3CL1 that is expressed in atherosclerotic plaques or endothelial cells (21). Spleen serves as a large reservoir of Ly-6C^hi^ monocytes during atherosclerosis (12, 13). Those Ly-6C^hi^ monocytes from spleen can rapidly emigrate to inflammatory sites and their inflammatory capacity is comparable to their counterparts from bone marrow or other reservoirs (22). The spleen, therefore, is serviced as major contributor to inflammatory macrophages and foam cell precursors in the growing atheromata. After splenectomy, the aortic root sections in mice contained fewer monocytes/macrophages and the plaques were smaller accordingly (23).

Mesenchymal stem cells (MSC), also known as multipotent mesenchymal stromal cells, are a cluster of well-established cells with non-hematopoietic, self-renewal, and multipotent differentiation properties (24). They can be isolated from different tissues, including bone marrow, umbilical cord, placenta, adipose tissue, and human gingiva (24–26). Recently, the anti-inflammatory and immunomodulatory effects of MSC on autoimmune and inflammatory diseases have been increasingly appreciated (27–29). Human gingiva-derived mesenchymal stem cells (GMSC) are a member of MSC and have been considered as a better source of MSC for their ease of isolation, homogeny, faster proliferation, stable characteristics, and stable karyotype (30, 31).

Of interest is a recent study showing that bone marrow-derived from mesenchymal stem cells (BM-MSC) can inhibit the formation of macrophage foam cells in ApoE^−/−^ mice (32). Research also has suggested that MSC act to restore endothelial function, reduce dyslipidemia, and stabilize plaques in atherosclerosis (33–35), but the underlying mechanisms are far from clear. Since our previous studies on GMSC also showed that GMSC possess considerable anti-inflammatory and immunomodulatory effects on immune cells (31, 36, 37), and macrophages play an important part in atherosclerosis, we supposed that GMSC might be able to modulate monocytes/macrophages and eventually alleviate atherosclerosis by this way. To elucidate the role of GMSC in atherosclerosis, we examined whether GMSC infusion reduced atherosclerosis in ApoE^−/−^ mice *in vivo*, as well as the effects of GMSC on macrophage foam cell formation and monocytes/macrophages activation and polarization. Our results suggest that GMSC infusion decreased inflammatory level and reduced plaque size and lipid deposition in ApoE^−/−^ mice *in vivo*, partly by inhibiting macrophage foam cell formation, modulating monocytes/macrophages activation and polarization *via* IDO and CD73 signals.

## Materials and Methods

### Reagents

Collagenase IV (C5138), phorbol 12-myristate 13-acetate (P8139), dispase II (D4693), lipopolysaccharides (L4391), ionomycin (I0634), oil red O (ORO) (O0625), l-1-methyltryptophan, and α, β-methylene ADP were obtained from Sigma-Aldrich. Recom-binant Human IL-4 (574004), IFN-γ (570206), IL-13 (571104), and Brefeldin A (420601) were purchased from Biolegend. Sodium poly-oxotungstate 1 (POM-1) was obtained from Tocris Bioscience. Human ox-LDL was obtained from Shanghai Lu Wen Biological Technology Co., Ltd. Antibodies were purchased from suppliers as follows: anti-GAPDH (G9545) was from Sigma-Aldrich; anti-CD36 (ab133625), anti-scavenger receptor A1 (SRA1) (ab183725), anti-ABCA1 (ab7360) or (ab18180), anti-CD68, goat anti-rabbit IgG H&L (HRP) (ab6721), goat anti-mouse IgG H&L (Alexa Fluor^®^ 488) (ab150113), goat anti-mouse IgG conjugated with Chromeo™ 546, and goat anti-rabbit IgG conjugated with Chromeo™ 546 (ab60317) were purchased from Abcam; fluorochrome-conjugated antibodies specific for human or mouse CD3, CD4, CD8, CD11b, IL-17A, IFN-γ, TNFα, IL-4, IL-10, IL-2, HLA-ABC, CCR2, Ly-6C, Gr1, HLA-DR (MHCII), CX3CR1, FOXP3, CD206, and CD86 were purchased from BD Biosciences, Biolegend, or eBioscience.

### Mice

Male apolipoprotein E knock out (ApoE^−/−^) mice (6–8 weeks old) were purchased from the Beijing Vital River Laboratory (the Beijing Vital River Laboratory Animal Technology Co., Ltd.,) and kept under standard laboratory conditions in the animal laboratory center of Sun Yat-sen University. Atherosclerosis was induced by feeding high fat diet (15% lard, 20% sugar, and 1.2% cholesterol) to ApoE^−/−^ mice for 10 weeks. GMSC of passage 2–3 were used. In each experiment, mice were randomly assigned into four groups: (1) *Model, n* = 6: mice consumed high fat diet and received 200 μL PBS per dose intravenously as control; (2) *Fibroblast, n* = 3: mice received 2 × 10^6^ human fibroblasts (a human cell line) in 200 μL PBS treatment per dose intravenously after consuming of high fat diet for 4 weeks, then received another dose at the seventh week of consuming high fat diet; (3) prevention group (*p-GMSC*), *n* = 5: mice received 2 × 10^6^ GMSC in 200 μL PBS treatment per dose intravenously before high fat diet, then received another two doses at the fourth and the seventh week of consuming high fat diet separately, with the same dose and the same cells from the same donor for the same mice; (4) Treatment group (*t-GMSC*), *n* = 7: mice received 2 × 10^6^ GMSC in 200 μL PBS treatment per dose intravenously after consuming of high fat diet for 4 weeks, then received another one dose at the seventh week of consuming high fat diet, with the same dose and the same cells from the same donor for the same mice. Successful intravenous injection was monitored by lack of extravasation at the injection site. No mice were died of these treatments. Then the mice consumed high fat diet for another 3 weeks before sacrificed. After 4% chloral hydrate (0.01 mL/g) was injected in abdominal cavity for anesthesia, the cardiac ventricle was perfused by PBS. The whole aortas from each group mice were dissected. Aortic roots from each group of ApoE^−/−^ mice were embedded in Tissue-Tek optimal cutting temperature compound (Changzhou Philas Instrument Co., Ltd., China) for sectioning. Fresh spleens and draining lymph nodes were collected and kept on ice for single living cell isolation. Experiments were repeated at least three times. All animals were treated according to National Institutes of Health guidelines for the use of experimental animals with the approval of the Sun Yat-sen University Third Affiliated Hospital Institutional Animal Care and Use Committee.

### Atherosclerosis

For the quantification of atherosclerotic plaque lesions in the vessels wall of ApoE^−/−^ mice, the whole aortas from each group mice were opened longitudinally and stained with ORO. Serial 10-μm-thick sections were cut from the aorta roots, every tenth section was subjected to ORO-staining, and photomicrographs were taken. The ORO-positive areas were analyzed with ImageJ software.

### Intracellular Cytokines

Fresh spleen cells and draining lymph node cells were isolated and stimulated with phorbol 12-myristate 13-acetate (PMA, 0.05 μg/mL) and ionomycin (0.5 μg/mL) for 1 h followed with brefeldin A (5 μg/mL) for 4 h at 37°C in a humidified tissue culture incubator with 5% CO_2_ and 95% O_2_. Then cells were collected, fixed, and permeabilized according to the manufacturer’s protocol of Intracellular Fixation & Permeabilization Buffer Set Kit (85-88-8824-00, eBioscience) before being stained with targeted FACS antibodies, such as CD4, CD8a, CD11b, Ly-6C, Gr1, IL-10, IL-17A, TNF-α, IFN-γ, and IL-4, followed by analyses with BD LSRFortessa Cell Analyzer.

### Confocal Microscopy

Frozen tissue samples were sectioned from the aorta at the origins of the aortic valve leaflets (5-µm thickness) in a cryostat. Then each sample was fixed for 20 min in 4% paraformaldehyde solution in PBS followed by permeabilization with 0. 1% Triton X-100 in PBS for 20 min. After washing three times with PBS, sections were incubated with 10% goat serum in PBS for 1 h at room temperature to block nonspecific binding. Then the sections were incubated overnight at 4°C with primary antibody either rabbit anti-SRA1 (1:200) (abcam), mouse anti-ABCA1 (1:200) (abcam), or mouse anti-CD68 (1:200) (abcam) in 2% BSA in PBS. After washing three times with PBS, the sections were incubated for 1 h in 2% BSA in PBS containing a 1:500 dilution of the appropriate secondary antibody, either a goat anti-rabbit IgG conjugated with Chromeo™ 546 or goat anti-mouse IgG conjugated with Alexa Fluor^®^ 488 (abcam) or goat anti-mouse IgG conjugated with Chromeo™ 546 (abcam). The sections were then incubated with Hoechst for 5 min to stain the cell nuclei after washing three times with PBS. The sections were again washed three times with PBS and mounted with glycerol followed by observing on a ZEISS LSM 710 Laser scanning confocal microscope or common fluorescence microscope. The confocal images were analyzed with ZEN 2009 Light Edition software.

### Cells Isolation and Culture

GMSC were obtained following the protocol as described previously (24). This study was carried out in accordance with the recommendations of the ethical review committee of clinical research of the Third Affiliated Hospital of Sun Yat-sen University. All human subjects gave written informed consent in accordance with the Declaration of Helsinki. Human tissue samples were obtained from discarded tissues of patients who had relatively healthy periodontium undergoing routine dental procedures and who provided informed consent in the Dental Division of the Third Affiliated Hospital at Sun Yat-sen University. Gingival tissues were treated aseptically and incubated overnight at 4°C with dispase II (2 mg/mL in PBS) followed with digestion by collagenase IV (4 mg/mL in PBS) at 37°C for 2 h after being minced into 1–3 mm^2^ fragments. Then the dissociated cell suspension was filtered through a 40-µm cell strainer (Falcon) and centrifuged to pellet cells. The cells were plated on a 10 cm petri dish with complete growth medium [MEM alpha (Gibco) supplemented with 10% fetal bovine serum (Gibco), 100 μg/mL penicillin/100 μg/mL streptomycin (Gibco), 100 µM MEM Non-Essential Amino Acids (Gibco), 550 µM 2-ME (Sigma-Aldrich), 10 mM Hepes, 1 mM sodium pyruvate, 2 mM l-glutamine], and cultured at 37°C in a humidified tissue culture incubator with 5% CO_2_ and 95% O_2_. After being cultured for 72 h, the non-adherent cells were removed. The plastic-adherent cells were passaged with 0.25% trypsin containing 1 mM EDTA when they reached a 80–90% confluent density, and subcultured in complete growth medium. We characterized GMSC by detecting their stem cell phenotypic markers and multipotent differentiation properties (24) (Figure S1 in Supplementary Material). Subcloning cultures were used to purify GMSC. Cells from the second to the third passages were used in the experiments.

THP-1 (TIB-202, ATCC), a human monocyte cell line, was a gift from Professor Yue-Qin Chen from the Key Laboratory of Gene Engineering of the Ministry of Education of Sun Yat-sen University. THP-1 was cultured in complete RPMI 1640 containing 10% fetal bovine serum (Gibco), 100 μg/mL penicillin/100 μg/mL streptomycin (Gibco), 100 µM MEM Non-Essential Amino Acids (Gibco), 550 µM 2-ME (Sigma-Aldrich), 10 mM Hepes (Gibco), 1 mM sodium pyruvate (Gibco), 2 mM l-glutamine (Gibco) at 37^°^C with 5% CO_2_. Cells were grown in suspension and diluted into 0.2–0.3 million cells/mL when the concentration reached 0.8–1.0 million cells/mL. Culture medium was changed every 2–3 days as necessary.

Bone marrow-derived macrophages (BMDMs) were isolated and induced as follows. Bone marrow cells from the hind legs of 6-week-old C57BL/6J mice were harvested and erythrocytes were lysed with Red Blood Cell Lysis Buffer (sigma). The CD11b^+^ cells were purified using magnetic isolation (Miltenyi Biotec). Then BMDMs differentiation was achieved by culturing the CD11b^+^ monocytes with MEM-α medium containing 10% FBS and 50 ng/mL M-csf for 5 days. BMDMs polarization toward M1 phenotype was accomplished by treatment with IFN-γ (20 ng/mL)/LPS (100 ng/mL) or M2 phenotype was accomplished by treatment with IL-4 (20 ng/mL)/IL-13 (20 ng/mL) for 48 h. All experiments were repeated at least three times with the similar results.

### Macrophages-GMSC Co-culture

The conditions for M1 macrophages, M2 macrophages, and macrophage foam cells induction are based on previous studies. In order to indirectly co-culture both macrophages and GMSC, THP-1 cells were cultured to M0 macrophages by stimulating with PMA (50 ng/mL) for 24 h in a 6-well plate. Indicated ratios of GMSC were cultured in the insert chambers in another 6-well plate overnight. Then these insert chambers were moved into the corresponding wells in which macrophages were cultured, and co-cultured with activated M0 macrophages in the presence of IFN-γ (20 ng/mL)/LPS (100 ng/mL) for M1 induction, IL-4 (20 ng/mL)/IL-13 (20 ng/mL) for M2 induction or ox-LDL (40ug/mL) for macrophage foam cell induction for 48 h. For macrophages and GMSC being co-cultured in direct cell–cell contact, GMSC were cultured overnight, then THP-1 was added and activated into M0 by PMA (50 ng/mL) stimulation for 6 h before adding IFN-γ/LPS, IL-4/IL-13, or ox-LDL for M1, M2, or foam cell induction, respectively for 48 h. All experiments were repeated at least three times with the similar results.

### Flow Cytometry

Cells were collected from culture plates or isolated from spleens and draining lymph nodes of sacrificed mice and resuspended in PBS. About 0.3 million cells per sample were stained with corresponding targeted FACS antibodies and incubated for 15 min at 4°C. For intracellular staining, cells were fixed and permeabilized before being stained with targeted FACS antibodies and incubated for 30 min at room temperature. Then cells were washed once, resuspended in PBS, and analyzed by BD LSRFortessa Cell Analyzer. FACS data were further analyzed on FlowJo 7.6.1.

### Western Blot Analysis

Cell extracts were prepared with whole cell lysis buffer (KeyGen BioTechonology, Nanjing, China) according to the manufacturer’s instructions. The protein concentrations were determined with the BCA Protein Assay Kit (Sangon BioTechonology, Shanghai, China). Equal amounts of cellular proteins (40 μg) were boiled for 5 min at 100°C and electrophoresed in a 10% gradient sodium dodecyl sulfate-polyacrylamide gel electrophoresis gel. Then proteins were transferred to polyvinylidene difluoride membranes (Millipore, USA). Nonspecific binding sites on the membranes were blocked with 5% BSA for 1–1.5 h at room temperature with gentle shaking. After that, membranes were reacted with primary antibody anti-CD36 (1:2,000) and anti-SRA1 (1:4,000) rabbit monoclonal antibody (abcam), anti-ABCA1 (1:500) rabbit polyclonal antibody (abcam), and anti-GAPDH (abcam) at 4°C in a shaker overnight. The membranes were then probed with a goat anti-rabbit IgG conjugated with horseradish peroxidase. The bands were visualized using an enhanced chemiluminescence kit (Millipore) and the images were captured by Tanon-5500 Chemiluminescent Imaging System (Tanon, China) and analysed with ImageJ software. All experiments were repeated at least three times with the similar results.

### The Tracking of GMSC *In Vivo*

GMSC were labeled with 5,6-carboxyfluorescein succinimidyl ester. Then the labeled cells were injected into C57BL/6 mice *via* tail vein injection. Each mouse received 2 × 10^6^ cells in 200 μl PBS. Every two mice were killed on 3, 7, 15, and 28 days after injection. Cells isolated from fresh lymph nodes and spleens of each mouse were stained fluorochrome-conjugated antibodies specific for human CD90 and analyzed by flow cytometry. Experiments were repeated one more time with the similar results.

### Serum Lipid Profiles and Cytokines Detection

Serum concentrations of total cholesterol, HDL-cholesterol, LDL/VLDL-cholesterol, and triglycerides were determined by colorimetric assay kit (abcam) according to manufacturer’s protocol. Serum TNF-α, IFN-γ, and IL-4 levels were determined in 3–4 animals per group by ELISA (Invitrogen) according to manufacturer’s protocol.

### Statistical Analysis

Statistical analysis was performed using GraphPad Prism (version 7.0). Data were analyzed by Student’s *t*-test in case of two groups or one-way ANOVA analysis in case of three and more groups in mice studies. Data are presented if not indicated elsewhere as mean ± SEM or mean ± SD. A value of *p* < 0.05 was considered to be statistically significant (*p < 0.05, **p < 0.01, ***p < 0.001, ****p < 0.001; NS, not significant).

## Results

### Infusion of GMSC Alleviates Atherosclerosis in ApoE^−/−^ Mice

**Figure 1:**
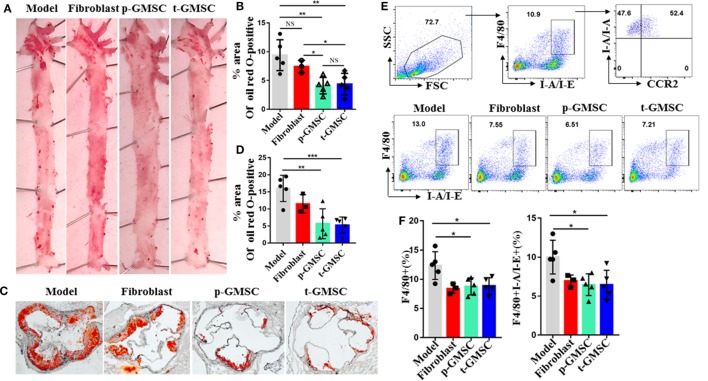
Systemic administration of human gingiva-derived mesenchymal stem cell (GMSC) accelerates atherosclerosis and reduce the number of macrophages in ApoE^−/−^ mice. Mice consuming a high fat diet pre-treated with GMSC (p-GMSC, *n* = 5), treated with GMSC 4 weeks after a high fat diet (t-GMSC, *n* = 5), treated with fibroblast (fibroblast, *n* = 3), or treated with PBS (model, *n* = 5) were sacrificed on 10 weeks after a high fat diet. **(A,B)** Representative images of Oil Red O (ORO) stained on the face aortic preparations. The bar graphs show the mean ± SD. **(C,D)** Representative images of ORO-stained aortic root sections. The bar graphs show the mean ± SD. Scale bar = 250 µm. **(E,F)** Splenocytes were stained with anti-F4/80, -I-A/I-E, and -CCR2 mAbs. Living cells were gated to determine percentages of F4/80^+^I-A/I-E^+^ macrophages. Representative dot plots of individual mice from each group were depicted. The bar graphs show the mean ± SD. *p < 0.05; **p < 0.01; ***p < 0.001. NS, *p* > 0.05.

To investigate the effect of GMSC on atherosclerosis development, ApoE^−/−^ mice were prevented or treated with GMSC before high fat diet (*p-GMSC*) or 4 weeks after high fat diet (*t-GMSC*). As a control, other two groups of mice were treated with PBS (*Model*) or fibroblast, respectively after 4 weeks of high fat diet. Fibroblasts were used as control because the morphology of these cells is similar to GMSC but not GMSC functionality. Since we can track living human GMSC even 28 days after cell infusion in immunocompetent mice (Figure S2 in Supplementary Material), we hypothesized that GMSC might be functional in animal model *in vivo*. Thus, we gave three doses of GMSC in *p-GMSC* group and two doses of these cells in *t-GMSC* as described in methods. After 10 weeks of high fat diet, we observed that serum concentrations of total cholesterol, HDL-cholesterol, and LDL/VLDL-cholesterol level showed no significant changes among different groups, while body weights and serum concentrations of triglyceride presented a significant decrease in ApoE^−/−^ mice received *p-GMSC* treatment (Figures S3A,B in Supplementary Material) when compared to model mice. Arterial plaques were presented mainly in the aortic arch in ApoE^−/−^ mice (Figure 1A). Interestingly, plaque areas in mice received GMSC in both prevention and treatment groups were significantly smaller than that in mice received fibroblast or PBS treatment and there was no significant difference between *p-GMSC* treatment and *t-GMSC* treatment (Figures 1A–D). Accordingly, we observed an increase in the expression ABCA1 and a decrease in the expression on SRA1 in the vessel walls of mice treated with GMSC when compared to mice treated with PBS (Figure S4 in Supplementary Material). Since macrophages are major immune cells involved in atherosclerosis, we analyzed the frequency and inflammatory phenotype of macrophages in various groups of mice. We observed that the frequencies of splenic and peripheral blood macrophages similarly and significantly decreased following *p-GMSC* or *t-GMSC* infusion (Figures 1E,F; Figure S5A in Supplementary Material). We also observed a drop on the frequency of macrophages in the draining lymph nodes of ApoE^−/−^ mice received *p-GMSC* or *t-GMSC* treatment though it was not significant (Figure S5B in Supplementary Material). The expression of HLA class II molecules (I-A/I-E; MHCII), an antigen presentation marker of inflammatory macrophages and contributing to LDL-derived antigen recognition by CD4^+^ T lymphocytes in atherosclerotic lesions (38, 39), also decreased correspondingly in *p-GMSC* and *t-GMSC* groups when compared to control groups (Figures 1E,F). Additionally, more than 50% of those MHCII^+^ macrophages are CCR2^+^ (Figure 1E), suggesting that GMSC also inhibit macrophage migration, since CCR2 is in favor of macrophage recruitment to plaque lesions. We also observed a decrease in the expression of M1 macrophages marked as F4/80^+^CD16/32^+^ in draining lymph node cells (40) (Figure S6A in Supplementary Material). We also observed that GMSC treatment, with or without GMSC pre-treatment before high fat diet, decreased the expression of CD68^+^ macrophages in aorta of ApoE^−/−^ mice (Figure S7 in Supplementary Material). These results together suggest that GMSC infusion alleviates atherosclerosis, which may partly *via* regulating the recruitment of inflammatory macrophages to plaque lesions, or decreasing inflammatory function of macrophage and maintaining lipid homeostasis of vessel wall cells.

### Infusion of GMSC Reduces the Expression of Inflammatory Ly-6C^hi^ Monocytes

**Figure 2:**
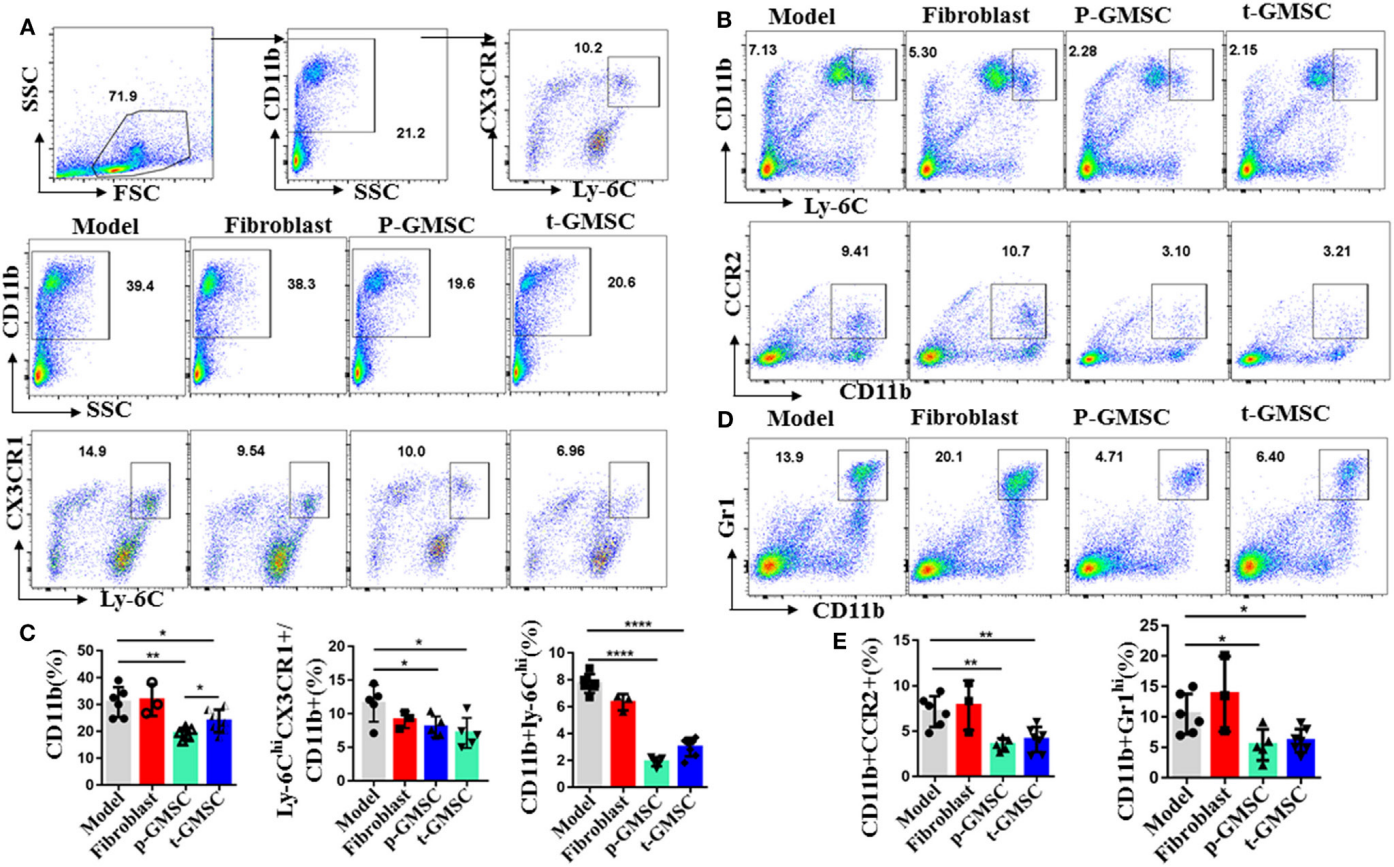
Human gingiva-derived mesenchymal stem cells (GMSC) decrease the expression of inflammatory Ly6C^hi^ monocytes in ApoE^−/−^ mice. The cells from spleen and draining lymph nodes of ApoE^−/−^ mice were stained with anti-CD11b, -Ly-6C, Gr1, CCR2, and -CX3CR1 mAbs. Living cells were gated to determine percentages of CD11b^+^, the subsets of Ly-6C^hi^CX3CR^+^ in CD11b^+^ cells, CD11b^+^Ly6C^hi^, CD11b^+^CCR2^+^, and CD11b^+^Gr1^hi^. **(A,B,D)** Representative dot plots of individual mice from each group were depicted. **(C,E)** Bar graphs show the mean ± SD (model, *n* = 6; fibroblast, *n* = 3; p-GMSC, *n* = 5; t-GMSC, *n* = 7). *p < 0.05; **p < 0.01; ***p < 0.001; ****p < 0.001.

Lesion local macrophages are end-effector cells which mainly differentiated from circulating Ly-6C^hi^ monocytes (23, 41). All these circulating monocytes are produced by hematopoietic progenitors in the bone marrow and spleen (42, 43). In the settings of hyperlipidemia, hematopoietic stem, and progenitor cells pro-gressively relocate from the bone marrow to the splenic red pulp, where they selectively expand Ly-6C^hi^ monocytes, leading to systematic monocytosis and disruption of the resolution of inflammation. To test the hypothesis that a high fat diet altered the repertoire of monocytes and that GMSC infusion had an effect on modulating these changes to alleviate atherosclerosis, we analyzed spleen cells, draining lymph node cells and peripheral blood cells from ApoE^−/−^ mice after consuming a high fat diet for 10 weeks with or without GMSC treatment. Compared with mice received fibroblast or PBS treatment, mice received *p-GMSC* or *t-GMSC* treatment which showed more than 40% decrease on the frequencies of CD11b^+^ monocytes in spleen (Figures 2A,C) and peripheral blood (Figure S6B in Supplementary Material). The *p-GMSC* treatment seems to be more efficient than *t-GMSC* treatment on CD11b^+^ monocytes reduction. Additionally, most of these CD11b^+^ cells are Ly-6C^+^ (Figure S8A in Supplementary Material). The subset of CD11b^+^Ly-6C^hi^, which is preferentially migrated to plaques and become lesional local inflammatory macrophages or contribute to plaque inflammation directly, also reduced simultaneously, especially in spleen (Figures 2B,C) and peripheral blood (Figure S6B in Supplementary Material) after *p-GMSC* or *t-GMSC* treatment. Correspondingly, the chemokine receptors which are in favor of their recruitment to plaque lesions like CX3CR1 and CCR2 on CD11b^+^ cells are also reduced after GMSC infusion (Figures 2A,C). Nevertheless, mice treated with GMSC also displayed a reduction on CD11b^+^Gr1^hi^ neutrophils in spleen (Figures 2D,E). Overall, our results indicate that GMSC treatment reduces the frequencies and the expression of chemokine receptors of inflammatory monocytes, especially Ly-6C^hi^ monocytes, which may help to shrink the reservoir and lower the migration of infiltrated inflammatory macrophages, attenuating inflammation and reducing plaque lesions in atherosclerotic ApoE^−/−^ mice.

### GMSC Modulate Cytokine Expression in Atherosclerotic Mice

**Figure 3:**
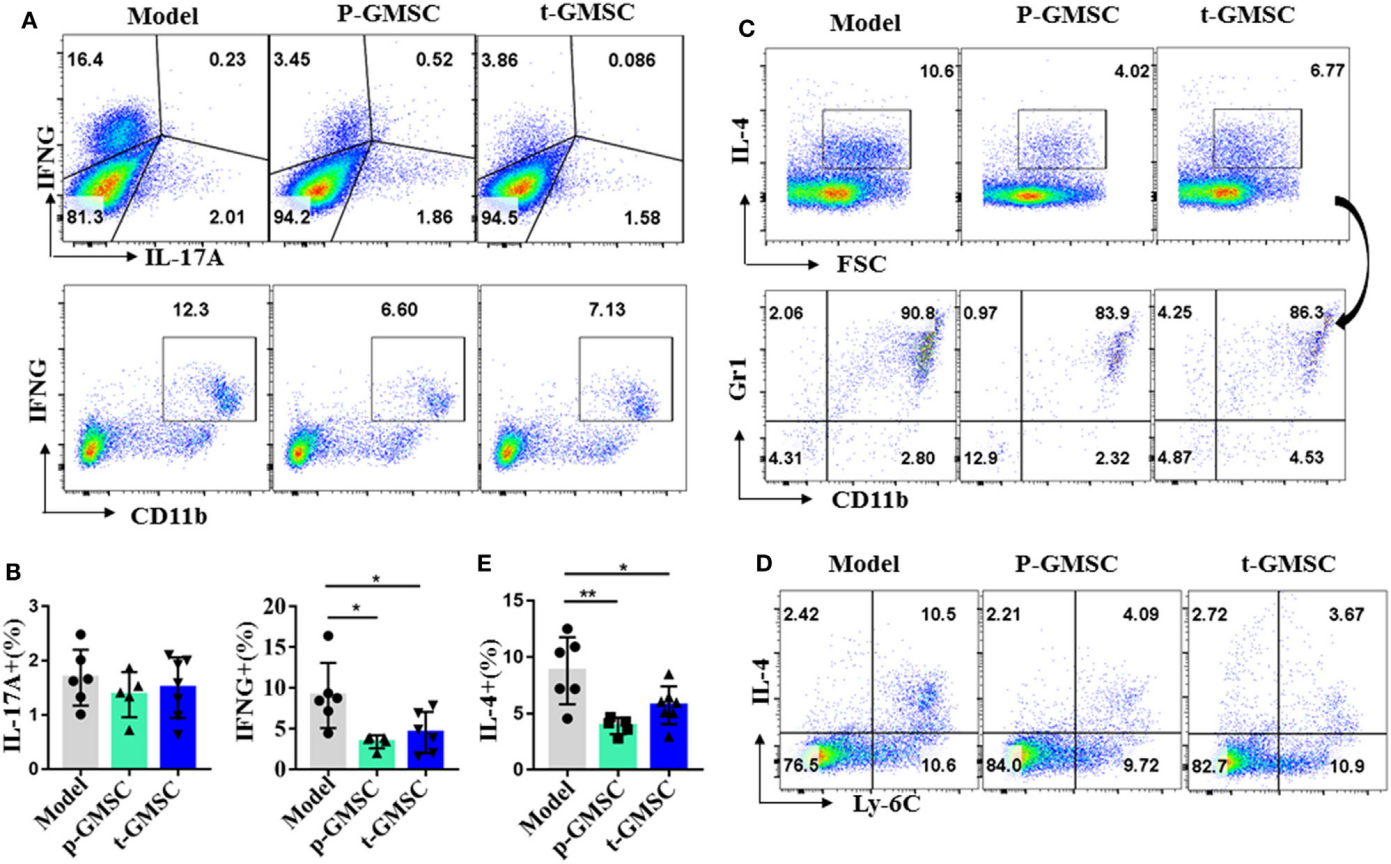
Effects of human gingiva-derived mesenchymal stem cells (GMSC) on the secretion of inflammatory cytokines in ApoE^−/−^ mice. Fresh spleen cells from ApoE^−/−^ mice were isolated and stimulated with phorbol 12-myristate 13-acetate (0.05 μg/ml) and ionomycin (0.5 μg/ml) for 1 h followed by blocking cytokines secretion with brefeldin A (1×) for 4 h. Anti-CD11b, Ly-6C, Gr1, and cytokines, such as IL-17A, TNF-α, IFN-γ, and so on were stained and analyzed with flow cytometry. **(A)** Representative dot plots of IL-17A, IFN-γ, and CD11b^+^IFN-γ^+^ expressing in spleen cells were depicted. **(C,D)** Representative dot plots of Ly-6C^+^IL-4^+^ and the subset of Gr1^+^CD11b^+^ in IL-4+ cells in spleen cells were depicted. **(B,E)** Bar graphs show the mean ± SD (model, *n* = 6; p-GMSC, *n* = 5; t-GMSC, *n* = 7). *p < 0.05; **p < 0.01.

Cytokines are key players during chronic inflammatory diseases like atherosclerosis (11). They affect endothelial permeability, the expression of adhesion molecules, lipid metabolism, and proliferation and migration of vessel intrinsic cells, which are all involved in atherosclerosis. IFN-γ is involved not only in early, but also in late stages of atherosclerosis. Advanced atherosclerotic lesions can be reduced in size and stabilized by IFN-γ inhibition, while administration IFN-γ accelerates atherosclerosis in ApoE^−/−^ mice (44, 45). After observing that GMSC treatment can reduce monocytosis and neutrophils, as well as inhibit the expression of inflammatory monocytes/macrophages in atherosclerotic mice, we further investigated whether GMSC infusion can alleviate inflammatory responses in these mice. To this end, the expression of intracellular cytokines, including TNF-α, IFN-γ, IL-10, IL-4, and IL-17A in spleen cells, draining lymph node cells and peripheral blood cells from ApoE^−/−^ mice were analyzed by flow cytometry. Compared with mice receiving PBS, mice receiving GMSC showed a significant reduction of IFN-γ and IL-4 in spleen cells (Figure 3), while there were no much changes in draining lymph nodes and peripheral blood cells (not shown). Additionally, instead of secreting by CD4^+^ T cells, most of the IFN-γ and IL-4 cytokines are secreted by CD11b^+^ cells (Figures 3A,C,D; Figure S9 in Supplementary Material). We also observed that most of those IL-4^+^ cells are Gr1^+^ and Ly-6C^+^ (Figures 3C,D). Since more than 90% of those increased CD11b^+^ cells are Ly-6C^+^, especially in mice without GMSC treatment, we may conclude that both IFN-γ and IL-4 are mainly secreted by CD11b^+^Ly-6C^+^ inflammatory cells. The secretion of IL-10 in spleen cells and draining lymph nodes remained unchanged (not shown). IL-17A, a pathogenic factor in atherosclerosis (46), was decreased slightly but no significant difference in spleen cells (Figure 3A) and draining lymph node cells (Figure S10A in Supplementary Material), while the expression of TNF-α in spleen and lymph node cells also remained unchanged (Figure S10B in Supplementary Material). We also detected the systemic inflammatory levels by detecting the serum concentrations of TNF-α, IFN-γ, and IL-4 in ApoE^−/−^ mice from different groups. We observed that all these cytokines presented a drop tendency with a significant decrease on IFN-γ level in mice received *p-GMSC* or *t-GMSC* treatment (Figure S11 in Supplementary Material). These results indicate that GMSC transplantation mainly decreases the pathological inflammatory responses by reducing the levels of pro-inflammatory cytokines, IFN-γ and IL-4, which are mostly secreted by CD11b^+^ monocytes.

### GMSC Inhibit Macrophage Foam Cell Formation and Modulate the Expression of ABCA1, CD36, and SRA1

**Figure 4:**
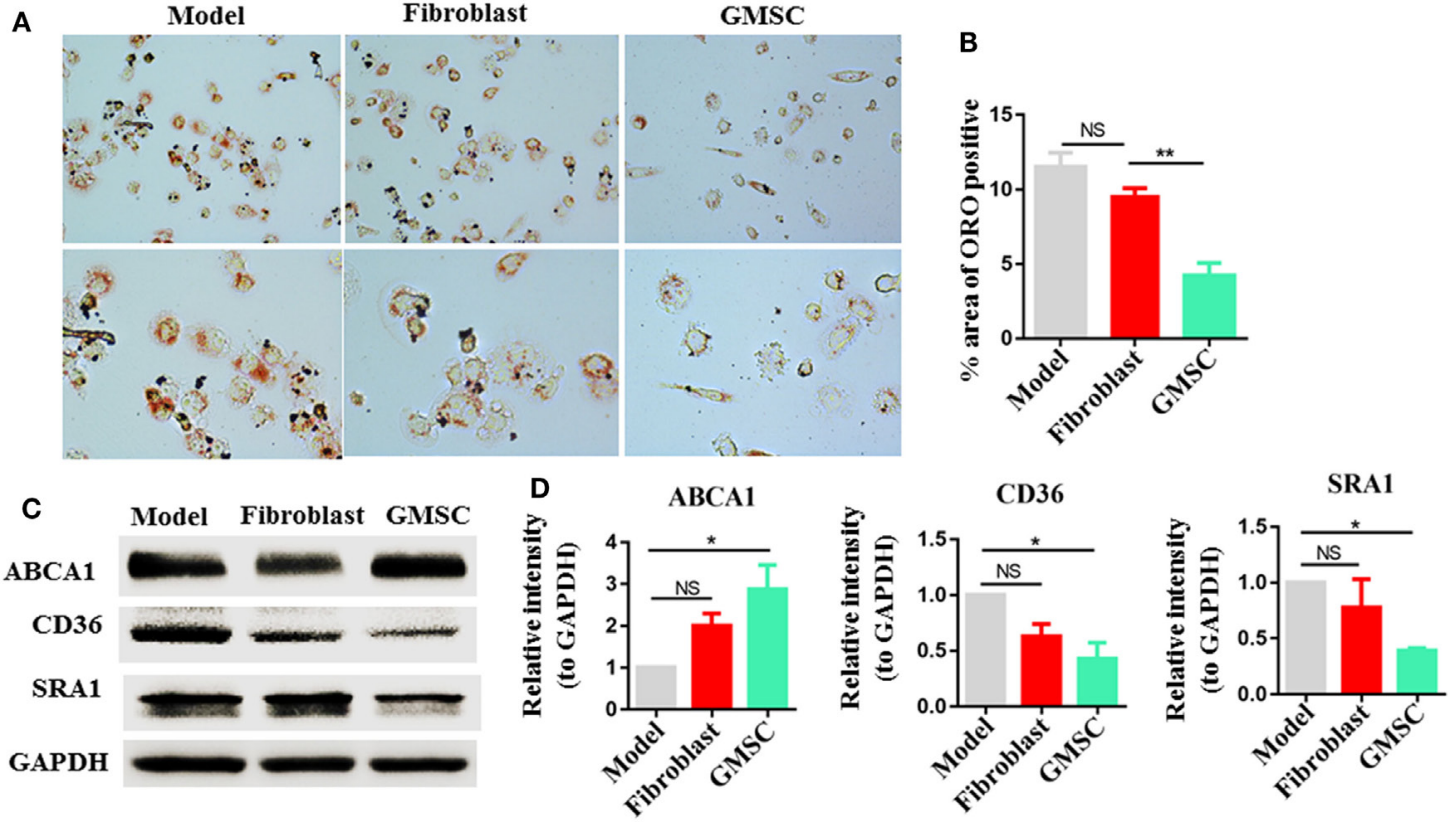
Effect of human gingiva-derived mesenchymal stem cells (GMSC) on the formation of macrophage foam cells. Macrophages were cultured in two conditions: model, macrophages were cultured in the presence of oxidized LDL (OX-LDL) for 48 h; fibroblast or GMSC, macrophages were co-cultured with GMSC or fibroblast in the presence of OX-LDL for 48 h. **(A,B)** Macrophages were stained with Oil Red O, representative photos from each group are depicted and the bar graphs show the mean ± SEM, *n* = 4. **(C,D)** Macrophages were subjected to Western blotting to determine the protein level of CD36, scavenger receptor A1 (SRA1), and ATP-binding cassette transporter A1 (ABCA1) and the bar graphs show the protein band intensity of ABCA1, CD36, and SRA1 normalized to GAPDH. Data are the representative of three independent experiments and presented as mean ± SEM. *p < 0.05; **p < 0.01; NS, *p* > 0.05.

The conversion of macrophages into foam cells is a critical step in the development of atherosclerosis and it runs through all stages of atherosclerosis (4, 10, 47). We have observed that GMSC infusion reduced atherosclerosis lesions, we further tried to figure out the influence of GMSC on foam cell formation. Macrophages activated from THP-1 were co-cultured with GMSC in indirect contact in the presence of ox-LDL for 48 h. Then ORO staining was performed on macrophages to evaluate the rates of foam cell formation. Compared to the macrophages cultured alone (model) or co-culture with fibroblast, macrophages co-cultured with GMSC showed about 50% decrease in foam cell formation (Figures 4A,B). Previous studies showed that scavenger receptors, such as CD36, SRA, and ABCA1, are critical in maintaining lipid homeostasis in macrophages (48–50). So we also determined whether GMSC functioned to modulate the expression of these receptors so as to inhibit foam cell formation. We found that GMSC treatment markedly inhibited the expression of CD36 and SRA1 while promoting the expression of ABCA1 (Figures 4C,D). These results indicate that GMSC inhibit foam cell formation and may affect lipid homeostasis in macrophages *via* modulating the expression of CD36, SRA1, and ABCA1.

### GMSC Suppress the Activation of M1 Macrophages and Promote Their Development Into the M2 Phenotype

Macrophages are a major driver of pathogenesis in atherosclerosis, by secreting inflammatory cytokines, such as TNF-α and interleukin-1β (IL-1β), and making communication with other immune cells, such as Th1 cells (51, 52). Our previous studies have shown that GMSC displayed immunomodulatory capacities similar to human BM-MSC while interacting with T cells (37). Since macrophage is an immune cell, we further explored the potential interplay between GMSC and macrophages.

**Figure 5:**
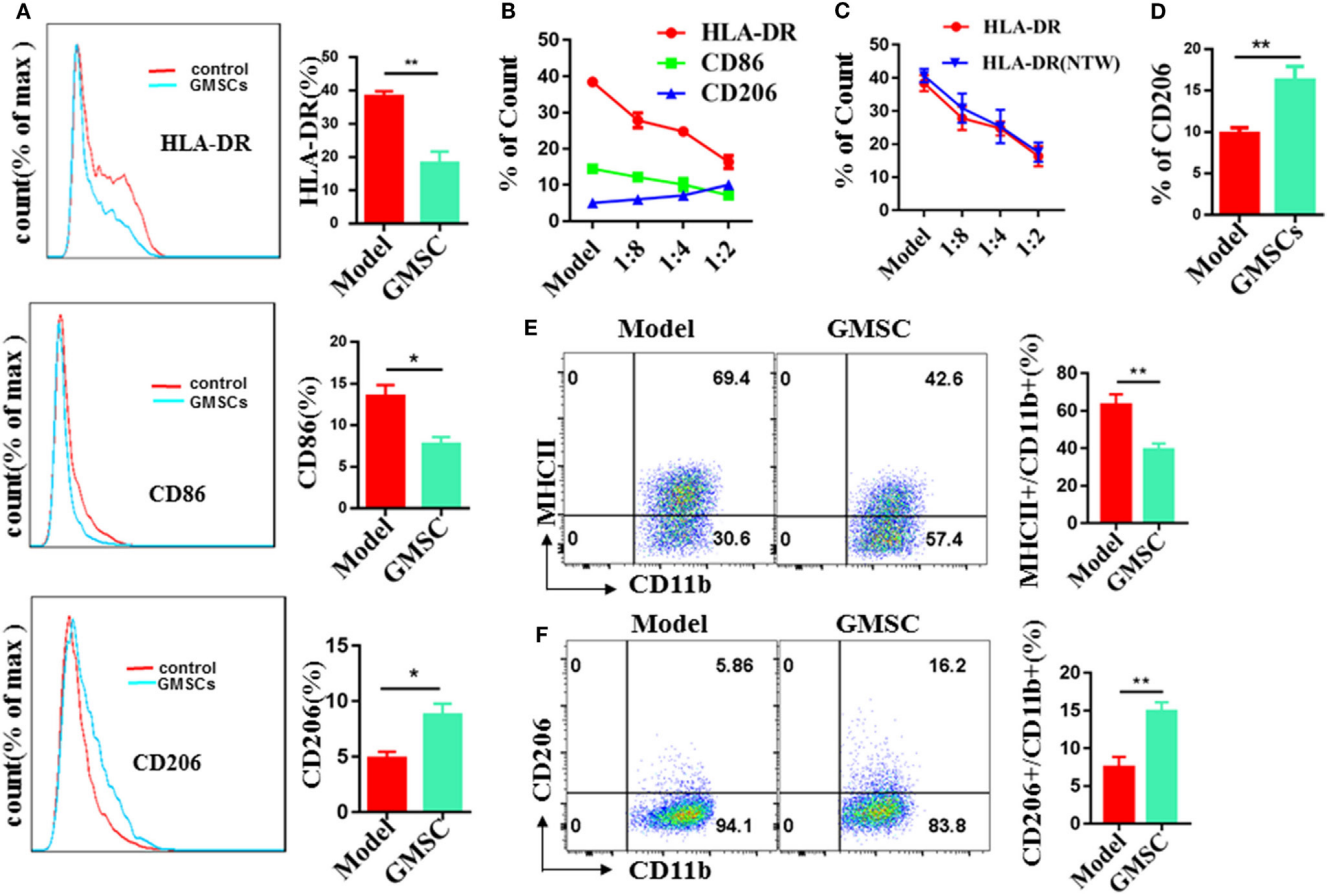
Human gingiva-derived mesenchymal stem cells (GMSC) inhibit the activation of macrophages and promote the expression of M2 macrophages. GMSC were co-cultured with THP-1 activated by phorbol 12-myristate 13-acetate (50 ng/mL) for 6 h followed by adding IFN-γ/LPS or IL-4/IL-13 for M1 or M2 induction respectively. After co-cultured for 48 h, cells were collected and the expressions of HLA-DR, CD86, and CD206 on macrophages were analyzed with flow cytometry. **(A)** GMSC and macrophages were co-cultured at a ratio of 1:2 (GMSC:THP-1) cell density in the presence of IFN-γ/LPS. **(B)** GMSC and macrophages were co-cultured in different proportions in the presence of IFN-γ/LPS. **(C)** GMSC and macrophages were co-cultured directly or indirectly at a ratio of 1:2 [GMSC:bone marrow-derived macrophages(BMDM)] cell density in the presence of IFN-γ/LPS. **(D)** GMSC and macrophages were co-cultured at a ratio of 1:2 (GMSC:THP-1) cell density in the presence of IL-4/IL-13. **(E)** GMSC and macrophages were co-cultured indirectly at a ratio of 1:2 (GMSC:BMDM) cell density in the presence of IFN-γ/LPS. **(F)** GMSC and macrophages were co-cultured indirectly at a ratio of 1:2 (GMSC:BMDM) cell density in the presence of IL-4/IL-13. Bar graphs show the mean ± SEM, *n* = 4. *p < 0.05; **p < 0.01.

THP-1 has been widely used as a cellular model to dissect the molecular mechanisms underlying monocyte-macrophage differentiation. So we chose THP-1 as the source of macrophages. We activated THP-1 to M0 macrophages by pulsing with PMA. Then, M0 macrophages were co-cultured with GMSC at a ratio of 2:1 (macrophages:GMSC) cell density for 48 h in the presence of LPS/IFN-γ for M1 macrophages induction or in the presence of IL-13/IL-4 for M2 induction. Moreover, macrophages were collected and determined on their expression of HLA-DR (53–55), CD86 (56–59) as M1 marker, and CD206 (M2) (60–62) as M2 marker. In the condition for M1 induction, macrophages co-cultured with GMSC showed 52.6% reduction in HLA-DR expression and 42.4% reduction in CD86 expression, while the expression of CD206 showed a significant increase when compared to macrophages cultured alone (Figure 5A). This effect was cell density-dependent (Figure 5B). The expression of HLA-DR showed no significant different regardless of GMSC and macrophages were co-cultured directly or indirectly, which supports that the modulation of GMSC on HLA-DR expression is depend upon the soluble cytokine(s) secretion instead of cell–cell contact (Figure 5C). We also observed that most of the CD206-positive macrophages were also positive for CD86 and HLA-DR. It was likely that CD206^+^ M2 macrophages may come from those already activated M1 macrophages, suggesting that GMSC treatment can mediate conversion of M1 into M2. In the condition for M2 induction, macrophages cultured in the presence of GMSC showed about 80% increase on the expression of CD206 (Figure 5D), while the expression of HLA-DR and CD86 stood still at a low level. In this condition, most of the macrophages positive for CD206 expressed no HLA-DR or CD86. These results suggest that CD86 and HLA-DR are not necessary when M2 macrophages are differentiated from M0 macrophages. We further used bone marrow-derived CD11b^+^ monocytes as the source of macrophages to validate these results and founded it had a similar result (Figures 5E,F). Either LPS/IFN-γ or IL-13/IL-4 had a significant effect on the immunomodulatory function of GMSC (Figure S12 in Supplementary Material). Overall, these results suggest that GMSC treatment can inhibit the expression of M1 macrophages and promote the expression of M2 macrophages that eventually changes the balance between M2/M1.

### IDO Signals and CD73 Signals Contribute to the Modulation of GMSC on Macrophage Differentiation

**Figure 6:**
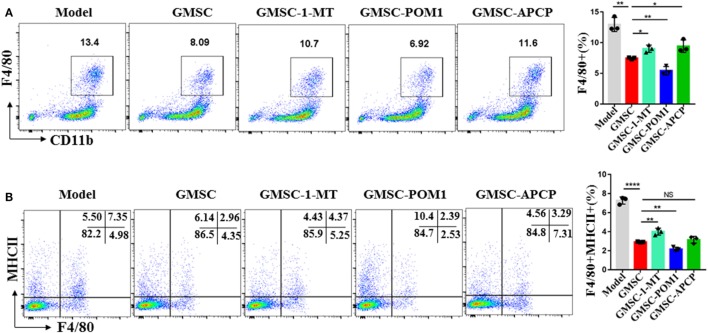
Human gingiva-derived mesenchymal stem cells (GMSC) modulate the differentiation of macrophages partly *via* CD73 and IDO signals. GMSC were pre-treated with α, β-methylene ADP (APCP, 100 µM), or sodium polyoxotungstate 1 (POM1, 100 µM) overnight and washed with complete medium. CD11b^+^ monocytes isolated from bone marrow of C57BL/6 mice and CD11b^+^ cells purified using magnetic isolation (Miltenyi Biotec) (purity: >95%) were added to co-cultured with GMSC with or without l-1-methyltryptophan (1-MT, 500 µM), co-cultured with GMSC, pretreated with POM1 or APCP in the presence of LPS(100 ng/mL) for 48 h, then suspending cells were collected and stained with fluorochrome-conjugated antibodies specific for mouse MHCII and F4/80 followed by analyzing with flow cytometry. **(A)** Representative dot plots of F4/80^+^ were depicted. **(B)** Representative dot plots of F4/80^+^MHCII^+^ were depicted. Bar graphs show the mean ± SD, *n* = 3. *p < 0.05; **p < 0.01; ***p < 0.001; ****p < 0.0001; NS, *p* > 0.05.

We have previous work prove that GMSC modulate immune cells responses *via* CD39/CD73/adenosine and/or IDO signals instead of HO-1, iNOS, TGF-β, IL-10, or PGE2 signals (37, 63). Indoleamine 2,3-dioxygenase (IDO) is a key enzyme in the kynurenine pathway of tryptophan metabolism. Its activity is linked with immunosuppression (64). Differentiated macrophages acquired the ability to suppress T cell proliferation *in vitro via* IDO signal and inhibition of IDO enhanced the elimination of virus-infected macrophages (64, 65). These studies suggested that IDO signal involves in modulating the function of macrophages. Both CD39 and CD73 are crucial for the degradation of ATP, AMP to generate adenosine to mediate immune suppression (66). After observing that GMSC treatment reduced inflammatory monocytes/macrophages in ApoE^−/−^ mice and modulated the activation and differentiation of macrophages, we speculated that CD39/CD73 and IDO signals might contribute to the modulation of GMSC on monocytes/macrophages. Regarding that monocytes/macrophages also express CD39/CD73, we pre-treated GMSC with CD73 inhibitor (α, β-methylene ADP, APCP) or/and CD39 inhibitor (POM1) before co-cultured with monocytes from bone marrow. We found that the levels of macrophages differentiated from monocytes under the stimulation of LPS showed a 40% decrease after being co-cultured with GMSC. Blocking the activity of CD73 but not CD39 GMSC partially restored the differentiation of macrophages (Figure 6A) and pretreated both inhibitors together did not resulted synergistic effect (data not shown). Similarly, addition of IDO inhibitor (1-MT) to the culture also partially restored macrophage differentiation (Figure 6A). We noted that addition of 1-MT to baseline culture without GMSC did not significantly change macrophage frequencies (not shown), suggesting the IDO activity is mainly related to GMSC. We also observed a similar result and mechanism on how GMSC regulate the expression of MHCII on macrophages (Figure 6B). However, addition the inhibitor of TGF-R1, IL-10, PGE2, iNOS, or HO-1 to the culture did not restore macrophage differentiation (data not shown).

## Discussion

Given increasing risk factors, such as obesity and physical inactivity, atherosclerotic disease currently remains a major cause of death. Lowering LDL-C in blood, mainly through lifestyle changes and statins, to decrease subendothelial lipid retention has been regarded as the most effective and direct way to prevent or treat atherosclerosis (67). However, cardiovascular risk reduction remains far from satisfactory and many patients cannot reach optimal LDL-C levels by statin treatment (68). Thus, additional therapies for effective lipid lowering to prevent atherosclerosis are needed.

Here, we show that GMSC treatment may be a promising strategy to alleviate atherosclerotic lesion development. First, we show here that GMSC treatment alleviates atherosclerosis, which may partly by reducing monocytosis and neutrophils in hyperlipemic mice considering of both of monocytosis and neutrophils contributing to the development of atherosclerosis (69). Those reduced cells after GMSC treatment are especially inflammatory Ly-6C^hi^ monocytes, which are thought to be a major source of lesion macrophages and contribute to lesion inflammation directly *via* secreting inflammatory cytokines, such as IFN-γ and IL-4.

It is noted that GMSC treatment decreased the frequency of inflammatory macrophage precursor cells, Ly-6C^hi^ monocytes. This is in line with a recent report that lipid lowering reduced inflammation in atherosclerosis through decrease of monocyte entry (70), although local proliferation of macrophages cannot be completely excluded in the pathogenesis of atherosclerosis (71, 72). Our observation highlights a potential advantage of GMSC in prevention and therapy in atherosclerosis, since it is likely that they affect both monocyte migration and local macrophage activation, proliferation, and differentiation in atherosclerosis.

We revealed that cytokines IFN-γ and IL-4 may be mainly secreted by CD11b^+^ cells, either Ly-6C^+^ or Gr1^+^ cells, GMSC treatment reduced their secretion which is consistent with the reduction of CD11b^+^ cells after GMSC injection. The proatherogenic effect of IFN-γ has been widely accepted. IFN-γ is supposed to promote M1 inflammatory macrophage polarization, inhibiting the secretion of IFN-γ may also contribute to inhibit M1 macrophage differentiation partly. With regarding to the influence of IL-4 in atherosclerosis and the primary source(s) of IL-4, the outcomes are controversial (73). Although IL-4, which is secreted by different kinds of cells including macrophages, Th2, mast, CD11b^+^ cell, and so on (74), is considered an anti-inflammatory cytokine and to promote the differentiation of M2 macrophages. It also plays an enhancing role in disease progression in some models of autoimmune diseases and atherosclerosis in animals (74–76). The proatherogenic role of IL-4 in atherosclerosis may partly explain by its influence on mononuclear cell recruitment *via* stimulating the expression of macrophage chemoattractant protein-1 (77) and its function to increase scavenger receptor expression by macrophages to increase the uptake of modified lipid and accelerate early lesion development (78).

The infiltration and activation of macrophages, as well as foam cell formation is a key step to initiate and promote atherosclerosis (10, 79). Accumulating evidence has demonstrated that there is a close link between inflammation, immunity, and lipid homeostasis in atherosclerosis (80). We have previously reported that GMSC can suppress T cells and mast cells, eventually preventing and treating inflammation and allergy diseases (31, 37, 63, 81, 82). We now provide a line of new evidence indicating that GMSC also suppress the differentiation, frequency, activation, and cytokine production of inflammatory monocytes and macrophages, leading to an alleviation of atherosclerosis in an animal model. It is interesting that the effects of GMSC on macrophages can be considered at least two ways. They not only suppress activation of inflammatory macrophages (M1) and foam cell formation, but also promote expression of anti-inflammatory macrophages (M2).

Our results display that GMSC treatment promotes the polarization of CD206^+^ M2 macrophages and these macrophages are reported to be characterized with increased phagocytic ability (36). We also observed that GMSC treatment decreases the expression of MHCII (antigen presentation marker) on macro-phages and decrease the expression of CD68^+^ (phagocytic marker) macrophages in aortic tissues of ApoE^−/−^ mice. A key event in atherosclerosis is a failure to resolve inflammation which involves suppressing influx of inflammatory cells and cleaning up of apoptotic cells effectively. Macrophages in atherosclerosis are a dynamic balance (3). Defective clearance of apoptotic cells by macrophages may lead to increased necrotic core formation in advanced lesions and aggravate atherosclerosis (83). However, increased recruitment of inflammatory macrophages also leads to failure of resolving inflammation and atherosclerotic progression. It is believed that decrease of macrophages appears to have an overall beneficial effect on early and advanced atherosclerosis, especially on early lesions, since the ability of phagocytes in these early lesions are efficient enough to clear apoptotic cells (84). It is likely that GMSC treatment alleviates atherosclerosis may partly *via* decreasing the recruitment of macrophages to lesions and improving phagocytosis function of local macrophages meanwhile.

We also explored the underlying mechanism(s) by which GMSC modulate the phenotype and function of macrophages and their relationship with atherosclerosis. It seems GMSC suppress monocytes/macrophages partly through IDO and CD73 signal pathways. This finding is consistent with previous reports on the role of GMSC in regulating T cells (63). Nonetheless, these pathways only play a partial role, implying that other unidentified molecules also involve in the immunosuppression on monocyte/macrophage-mediated diseases that are under investigation by author’s group now.

Studies have shown that age and age-associated conditions can impair the properties and functions of MSC (85–88). Since atherosclerosis is a multifactorial chronic disease which is usually accompanied by age and age-related diseases, and the vast majority of patients that may benefit from GMSC therapies in atherosclerosis are elderly individuals, our future studies will also focus on clarifying whether the functions of GMSC are affected by age and age-associated diseases as well as the underlying molecular mechanism(s) by which aged-GMSC function may weaken so as to maximize the therapeutic effects of GMSC in atherosclerosis.

In conclusion, we have shown that GMSC treatment alleviates atherosclerosis, partly through decreasing monocytosis and modulating the activation and differentiation of macrophages in ApoE^−/−^ mice. GMSC also affect lipid metabolism through restraining inflammation and modulating lipid metabolism related receptors. In addition, GMSC infusion not only prevents, but also treats atherosclerosis. These findings further support the notion that GMSC, a unique population of MSC with phenotypic and functional similarities to other MSC, may be a promising cell source for stem cell-based therapies in atherosclerosis and other inflammatory diseases.

## Ethics Statement

This study was carried out in accordance with the recommendations of “the ethical review committee of clinical research of the Third Affiliated Hospital of Sun Yat-sen University” with written informed consent from all subjects. All subjects gave written informed consent in accordance with the Declaration of Helsinki. The protocol was approved by the “Center for Clinic Immunology.” This study was carried out in accordance with the recommendations of “Sun Yat-sen University for the Use and Care of Animals.” The protocol was approved by the “Center for Clinic Immunology.”

## Author Contributions

SZ, XQ, XZ, and HT conceived and designed the research; XZ, WL, CS, FH, JY, JW, JD, ZL, YL, QA, and WS performed the research; XZ, SZ, and NO analyzed results and wrote the paper. All the authors agree to be accountable for the content of the work.

## Conflict of Interest Statement

The authors declare that the study was conducted in the absence of any commercial or financial relationships that could be construed as a potential conflict of interest.

## Abbreviations

GMSC, human gingiva-derived mesenchymal stem cells; MSC, mesenchymal stem cells; ox-LDL, oxidized LDL; SRA1, scavenger receptor A1; ABCA1, ATP-binding cassette transporter A1; AT-MSC, cardiac adipose tissue-derived mesenchymal stromal cells; BM-MSC, bone marrow-derived from mesenchymal stem cells; ApoE^−/−^, apolipoprotein E knock out; SDS-PAGE, sodium dodecyl sulfate-polyacrylamide gel electrophoresis; PMA, phorbol 12-myristate 13-acetate; BMDMs, bone marrow-derived macrophages.
