# Supplementary material for: Human Gingiva-Derived Mesenchymal Stem Cells Modulate Monocytes/Macrophages and Alleviate Atherosclerosis
Source: Front Immunol. 2018 Apr 30;9:878. doi: 10.3389/fimmu.2018.00878 (PMC5937358; doi:10.3389/fimmu.2018.00878)
Supplement: Supplementary file 1 [file Image_1.PDF]

Supplementary Material

Human Gingiva-Derived Mesenchymal Stem Cells Modulate Monocyte/Macrophages and Alleviate Atherosclerosis

Ximei Zhang[1,2], Feng Huang[2], Weixuan Li[3], Jia Yuan[4], Julie Wang[5], Hongmei Tan[3], Xiaoxian Qian[1,*], Nancy Olsen[5], Song Guo Zheng[5,*]

***Correspondence:**
Song Guo Zheng, MD, PhD or Xiaoxian Qian, MD, PhD, **szheng1@hmc.psu.edu, or xiaoxianq@qq.com.**

Figure S1

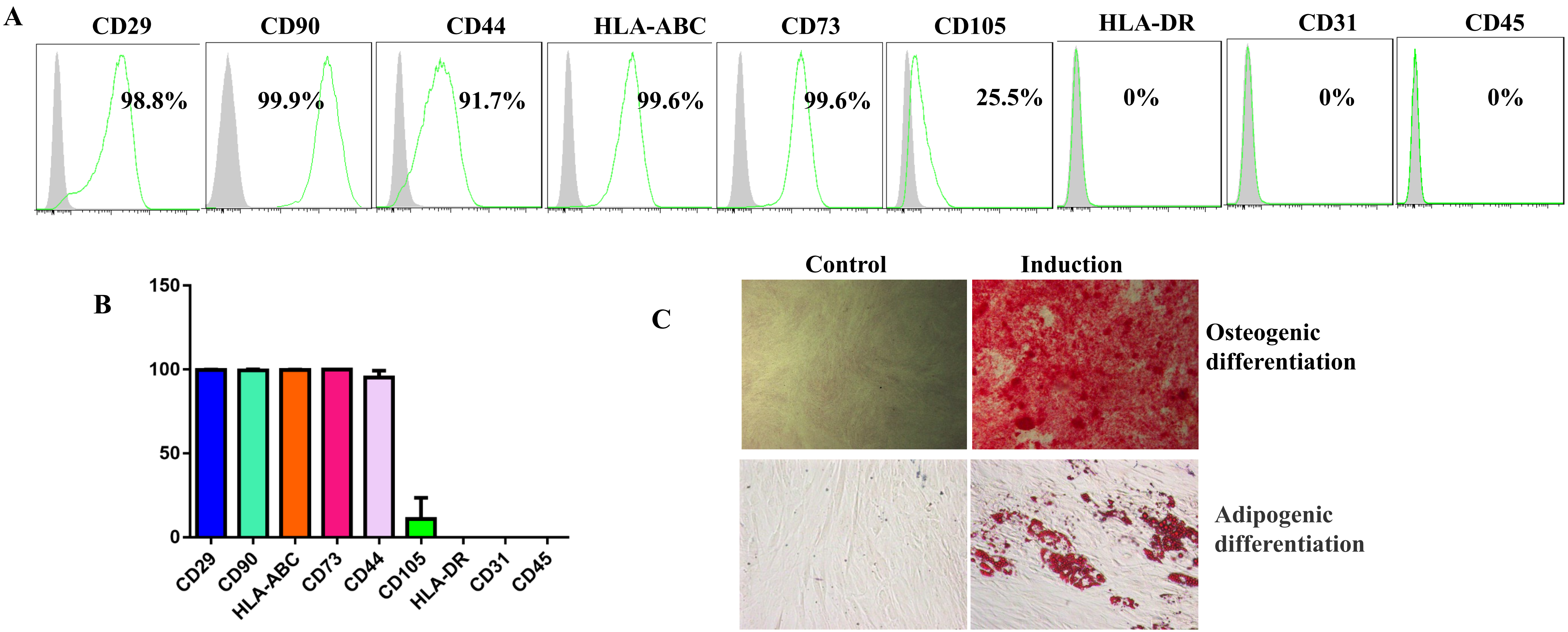

Figure S1. Phenotypic markers and differentiation function ofGMSCs. (A,B) Expression of cell surface markers on GMSCs analyzed by flow cytometry, the Bar graphs show the Mean ± SEM. (C) Osteogenic differentiation(Alizarin Red S staining) and adipogenic differentiation(Oil Red O staining) of GMSCs.

Figure S2

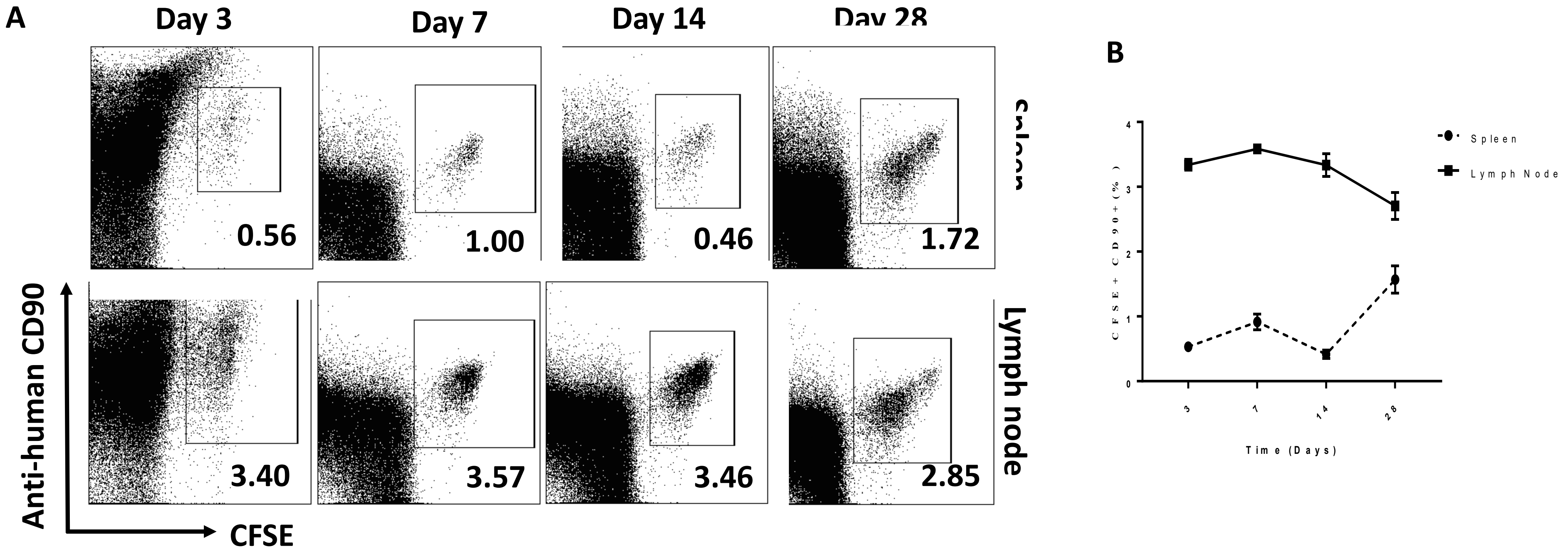

Figure S2. Intravenous injected GMSCs migrated to spleen and lymph nodes.
GMSCs labeled with 5,6-carboxyfluorescein succinimidyl ester (CFSE) were injected into C57BL/6 mice *via tail vein* and the distribution of GMSCs was detected by flow cytometry at different time points post-injection. (**A**) Representative dot plots of individual mice showed that *i.v.* injected GMSCs could home to lymph nodes and spleens in a large percentage, regardless of a lower percentage at different time points. (**B**) Statistic chart for (**A**). Each time point included two mice and mean ± SD of each time point in different organ were shown. Experiments were repeated twice with the similar results.

Figure S3

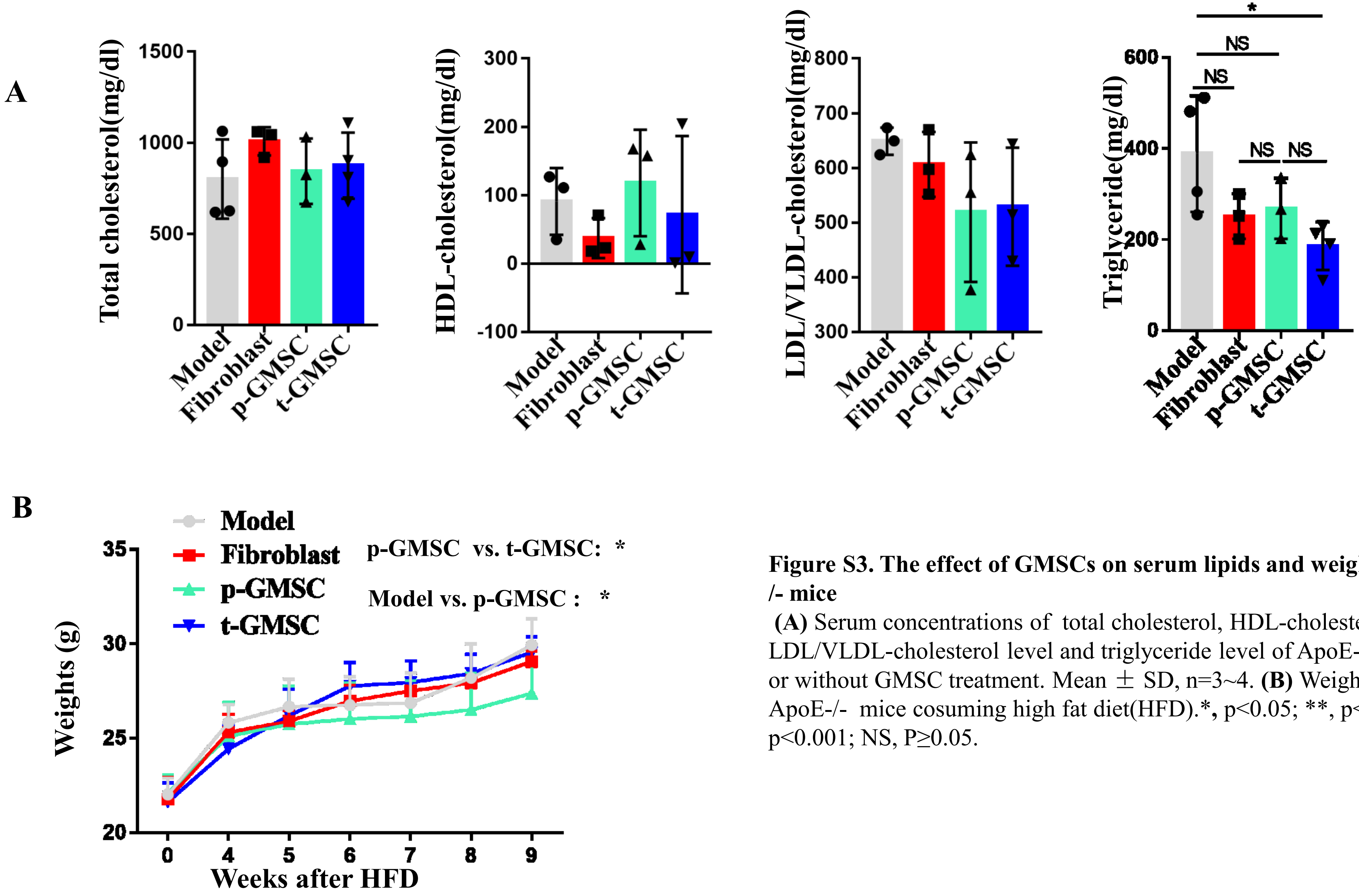

Figure S3. The effect of GMSCs on serum lipids and weights of ApoE-/- mice

(A) Serum concentrations of total cholesterol, HDL-cholesterol, LDL/VLDL-cholesterol level and triglyceride level of ApoE-/- mice with or without GMSC treatment. Mean ± SD, n=3~4. **(B)** Weight changes of ApoE-/- mice cosuming high fat diet(HFD).*, $p<0.05$; **, $p<0.01$; ***, $p<0.001$; NS, $P\geq0.05$.

Figure S4

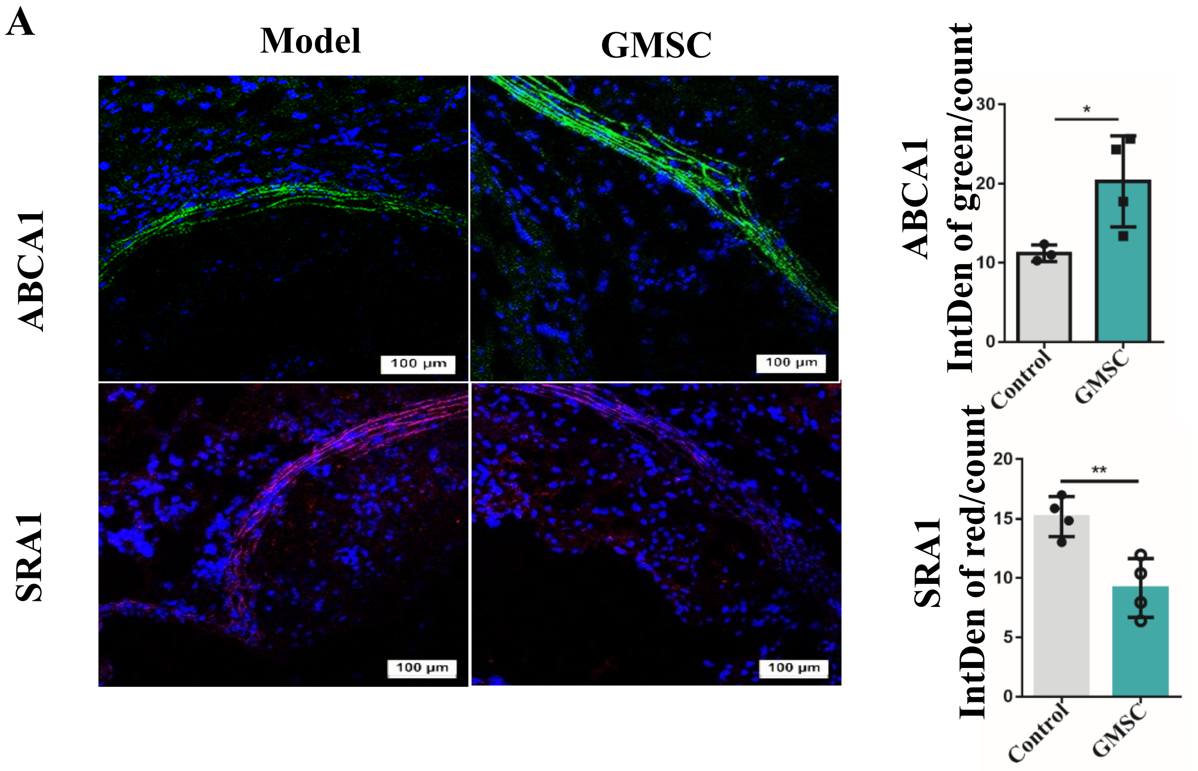

Figure S4. Systemic administration of GMSCs regulated the expression of ABCA1 and SRA1 in vessel wall of ApoE-/-mice.

(A): Cryosections were incubated with the primary antibody anti-SRA1(1:200) and anti-ABCA1(1:200) followed with incubation with a Goat anti-rabbit IgG conjugated with Chromeo™ 546(red) and Goat anti-mouse IgG conjugated with Alexa Fluor® 488(green) secondary antibody respectively. Cell nuclei were counterstained with Hoechst (blue). The slides were mounted with glycerol and the expression of ABCA1(green) and the expression of SRA1(red) w observed on a ZEISS LSM confocal microscope. Representative sections from three mice in each group are shown. The Bar graphs show the Mean ± SEM, n=3. *, $p<0.05$; **, $p<0.01$; ***, $p<0.001$.

Figure S5

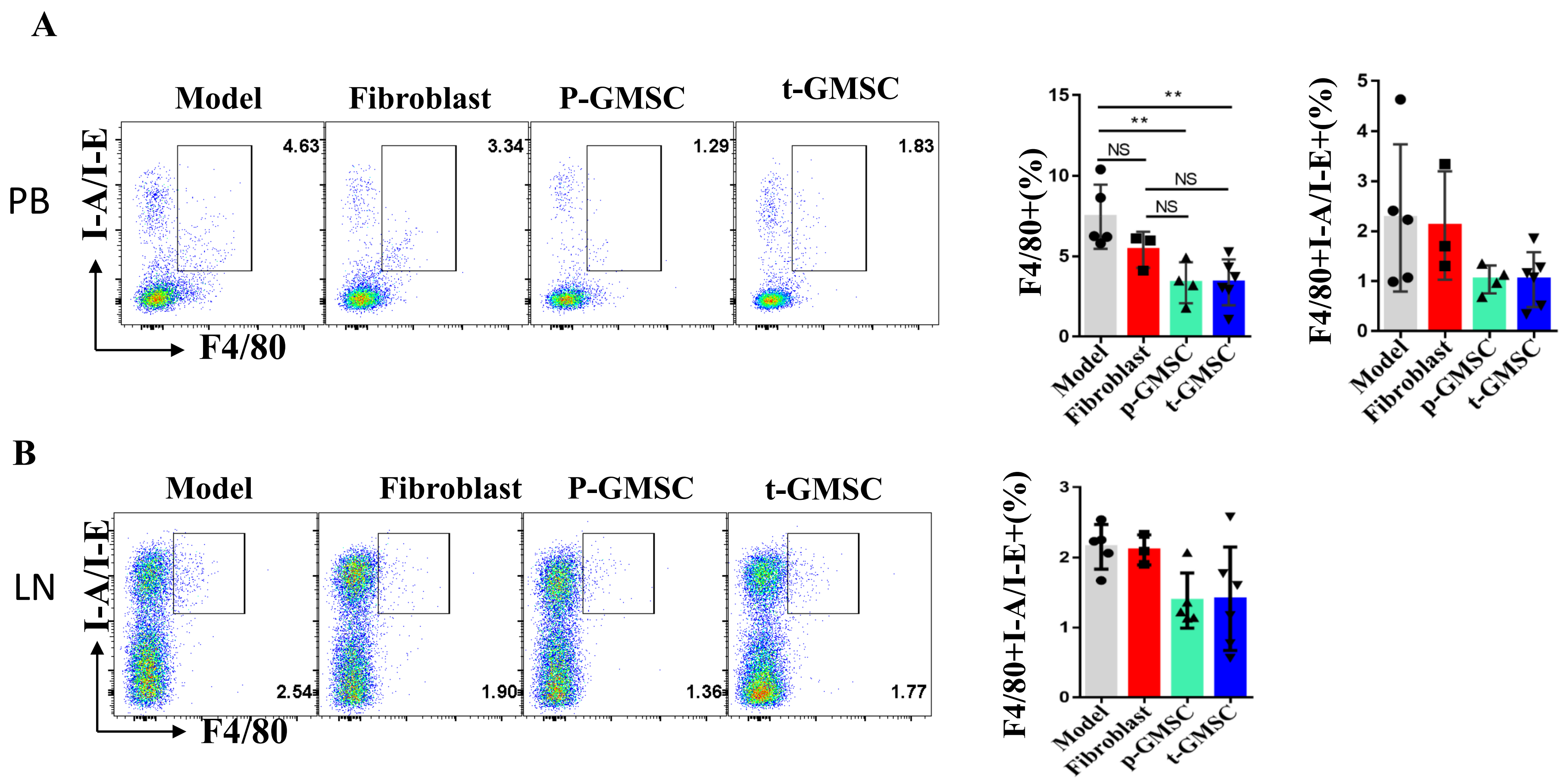

Figure S5. Single cells from peripheral blood cells**(PB, A)** or draining lymph nodes**(LN, B)** of ApoE$^{-/-}$mice were stained with anti-F4/80 and –I-A/I-E mAbs. Living cells were gated to determine percentage of F4/80+ or F4/80+I-A/I-E+ macrophages**.** Representative dot plots of individual mice from each group were depicted. The Bar graphs show the Mean ± SD(Model, n=5; Fibroblast, n=3; p-GMSC, n=4; t-GMSC, n=6). *, $p<0.05$; **, $p<0.01$; ***, $p<0.001$.

Figure S6

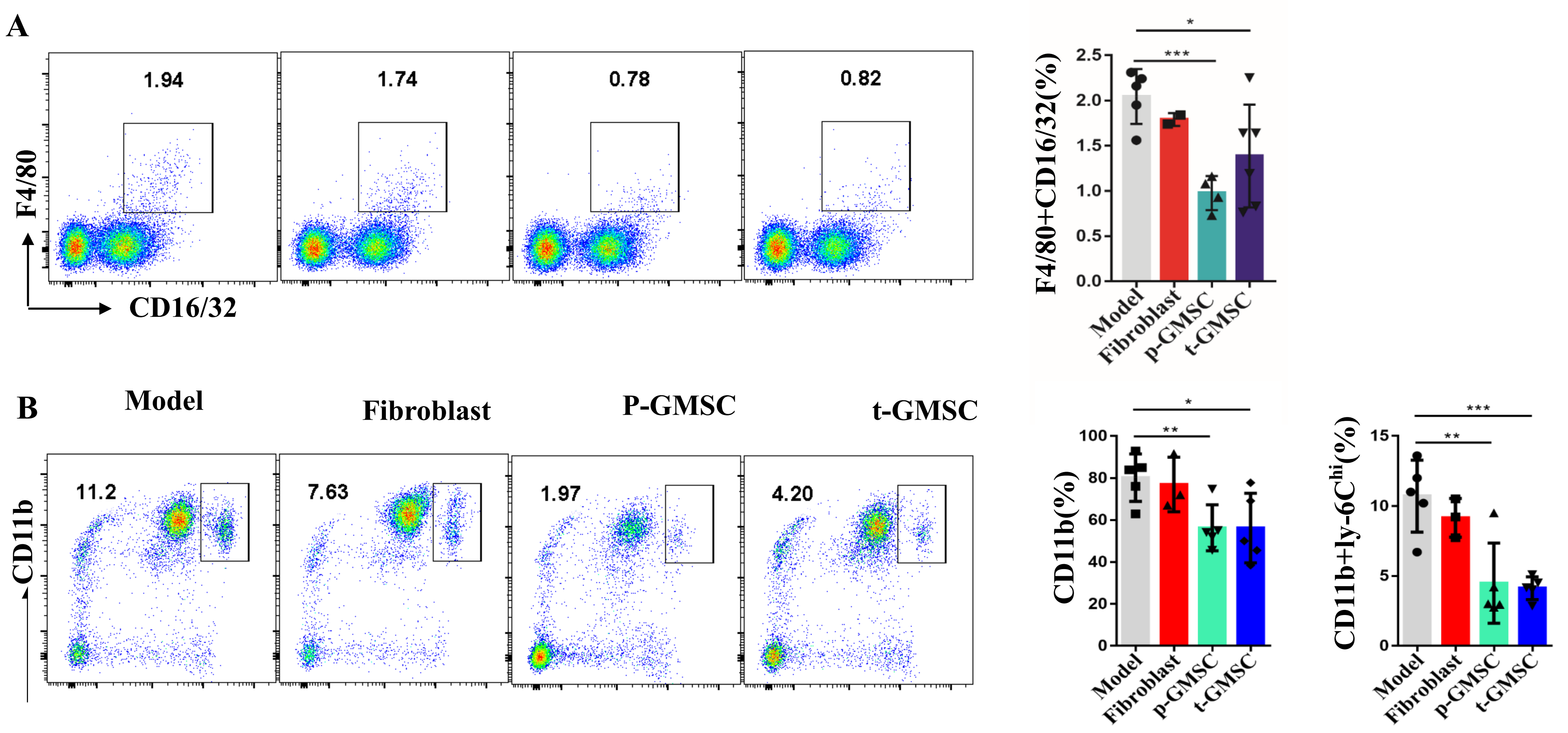

Figure S6. Single cells from draining lymph nodes**(A)** or peripheral blood cells**(B)** of $ApoE^{-/-}$mice were stained with anti-F4/80, -CD16/32, -CD11b and Ly-6C mAbs. Living cells were gated to determine percentage of F4/80+ CD16/32+ macrophages or CD11b+ Ly-$6C^{hi}$ monocytes**.** Representative dot plots of individual mice from each group were depicted. The Bar graphs show the Mean ± SD(Model, n=5; Fibroblast, n=3; p-GMSC, n=4; t-GMSC, n=5). *, $p<0.05$; **, $p<0.01$; ***, $p<0.001$.

Figure S7

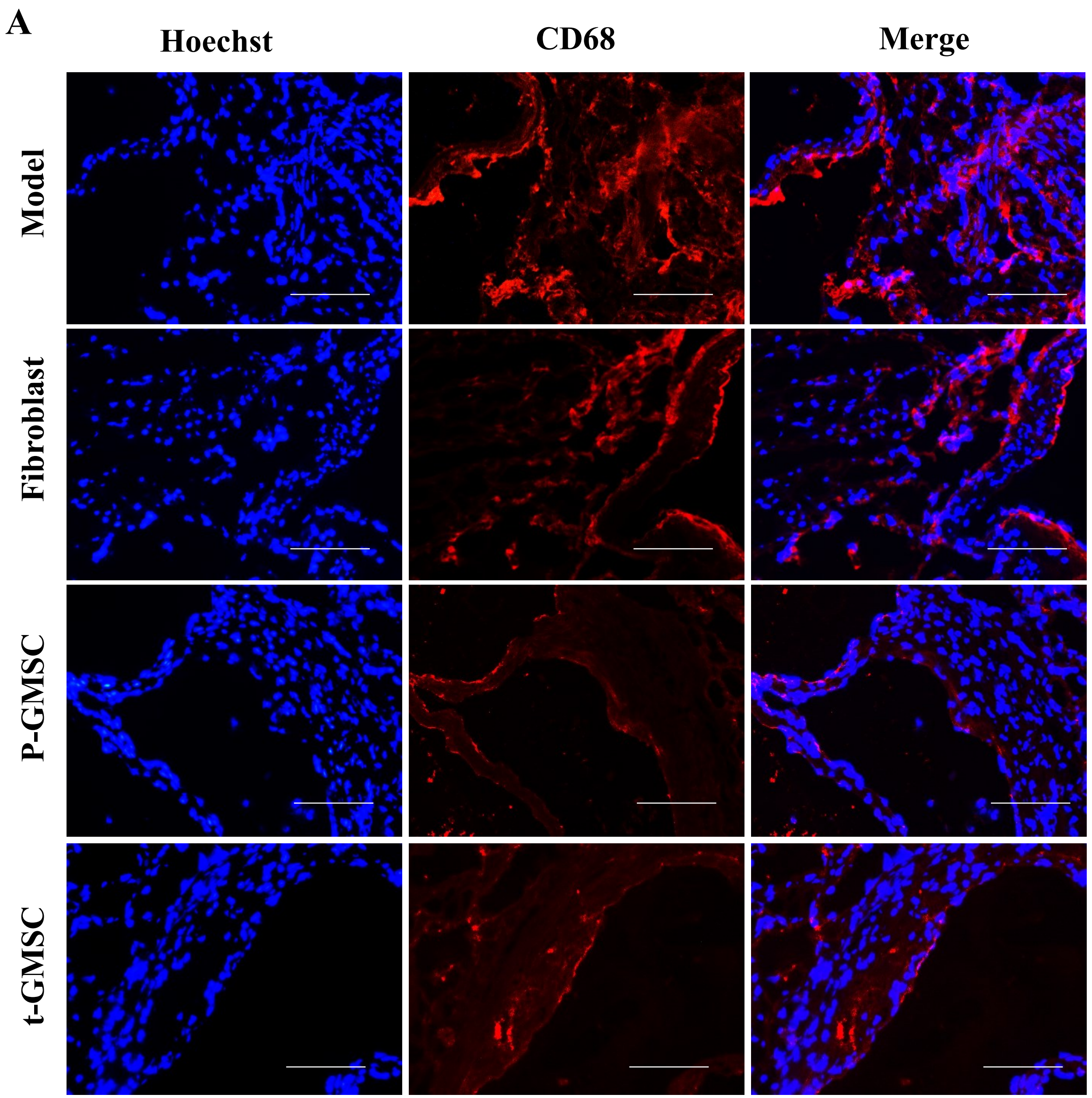

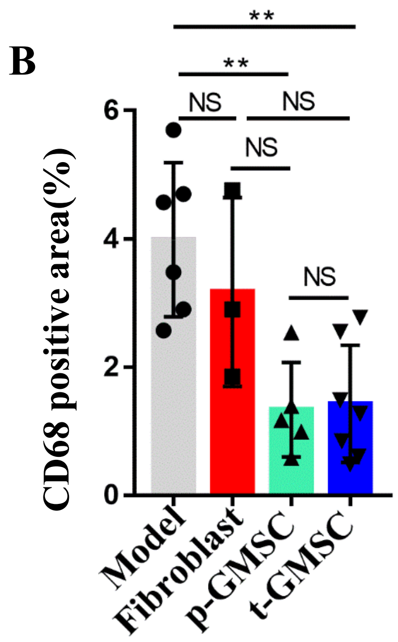

Figure S7. GMSCs treatment decreased the expression of CD68+macrophages in aortic tissues of ApoE-/- mice.

(A)Representative images of transverse aortic sinus frozen sections stained with anti-CD68(Red) and Hoechst(blue,nuclei) of ApoE-/- mice consuming a HFD for 10 weeks(scale bar, 50μm). (B) CD68-positive area(%), the bar graph show the Mean ± SD(Model, n=6; Fibroblast, n=3; p-GMSC, n=5; t-GMSC, n=7). *, $p<0.05$; **, $p<0.01$; ***, $p<0.001$; NS, $p\geq0.05$.

Figure S8

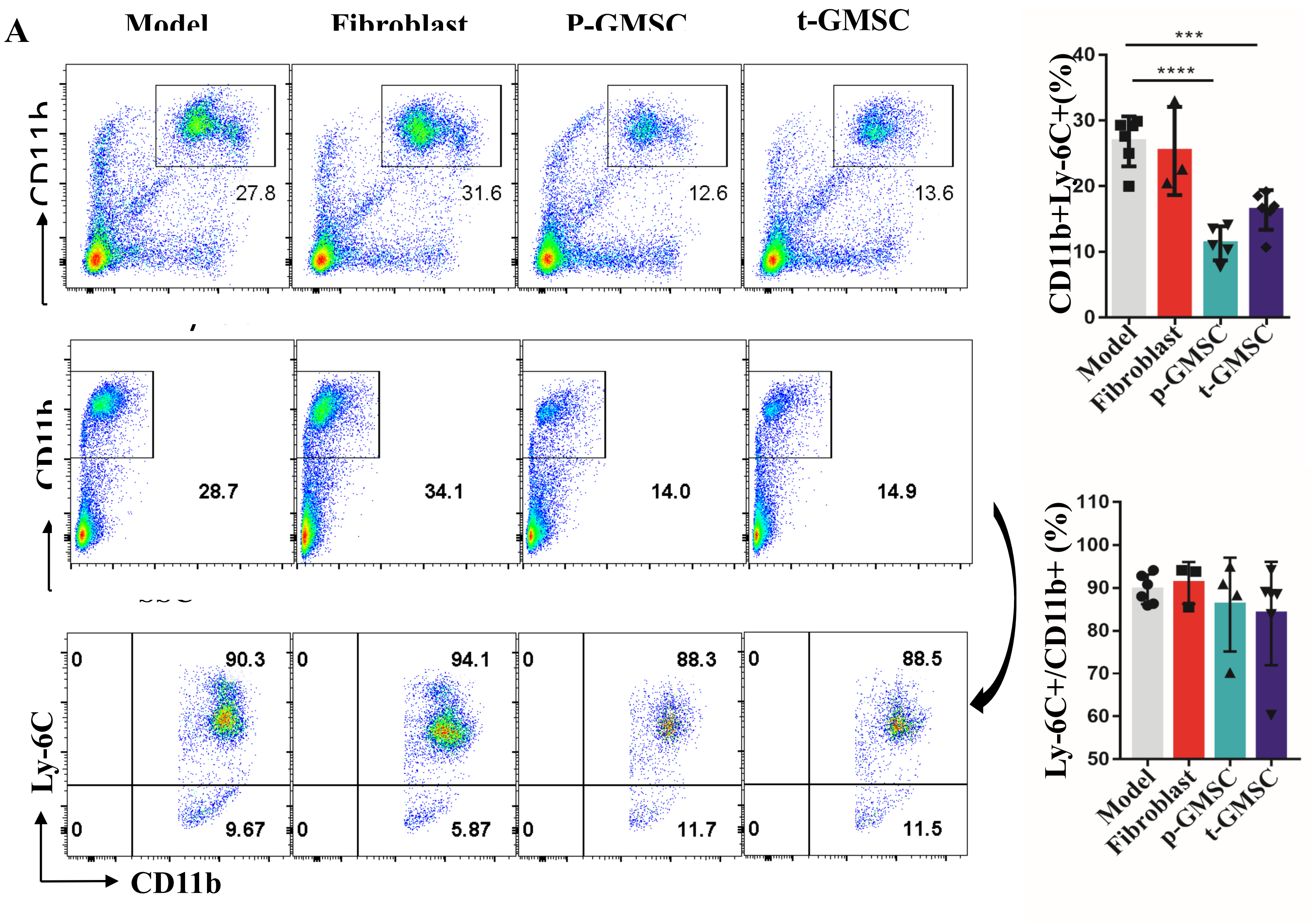

Figure S8. Single cells spleens of ApoE$^{-/-}$mice were stained with anti-CD11b and Ly-6C mAbs. Living cells were gated to determine percentage of CD11b+, CD11b+ Ly-6C+ or Ly-6C+/11b+ cells. Representative dot plots of individual mice from each group were depicted. The Bar graphs show the Mean ± SD (Model, n=6; Fibroblast, n=3; p-GMSC, n=5; t-GMSC, n=5). *,p<0.05; **, p<0.01; ***, p<0.001.

Figure S9

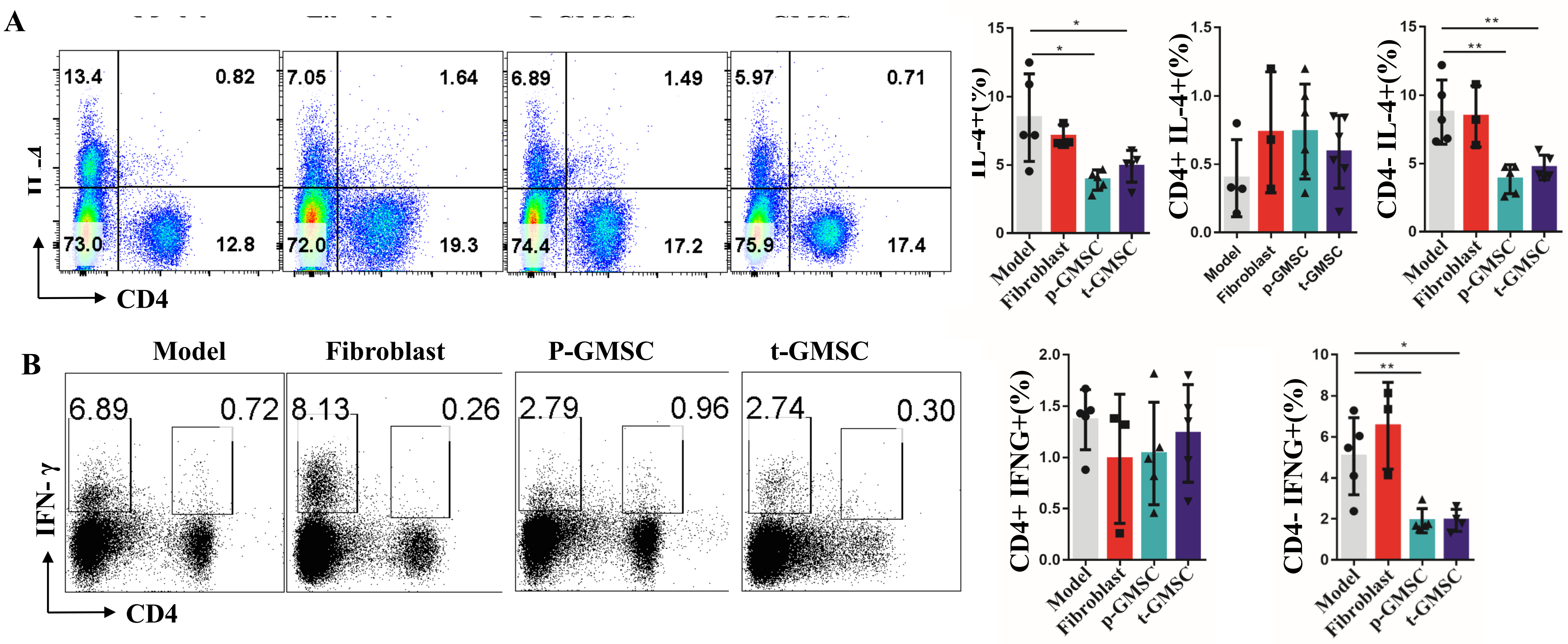

Figure S9. Fresh spleen cells of ApoE$^{-/-}$mice were isolated and stimulated with phorbol12-myristate13-acetate (PMA, 0.05ug/mL) and ionomycin(0.5ug/mL) for 1h followed with brefeldin A (5ug/mL) for 4h at 37℃ in a humidified tissue culture incubator with 5% CO_2 and 95% O_2. Then cells were stained with anti-CD4, IL-4 and IFN- γ mAbs. Living cells were gated for further analysis**.** Representative dot plots of individual mice from each group were depicted. The Bar graphs show the Mean ± SD(Model, n=5; Fibroblast, n=3; p-GMSC, n=5; t-GMSC, n=5).*, p<0.05; **, p<0.01; ***, p<0.001.

Figure S10

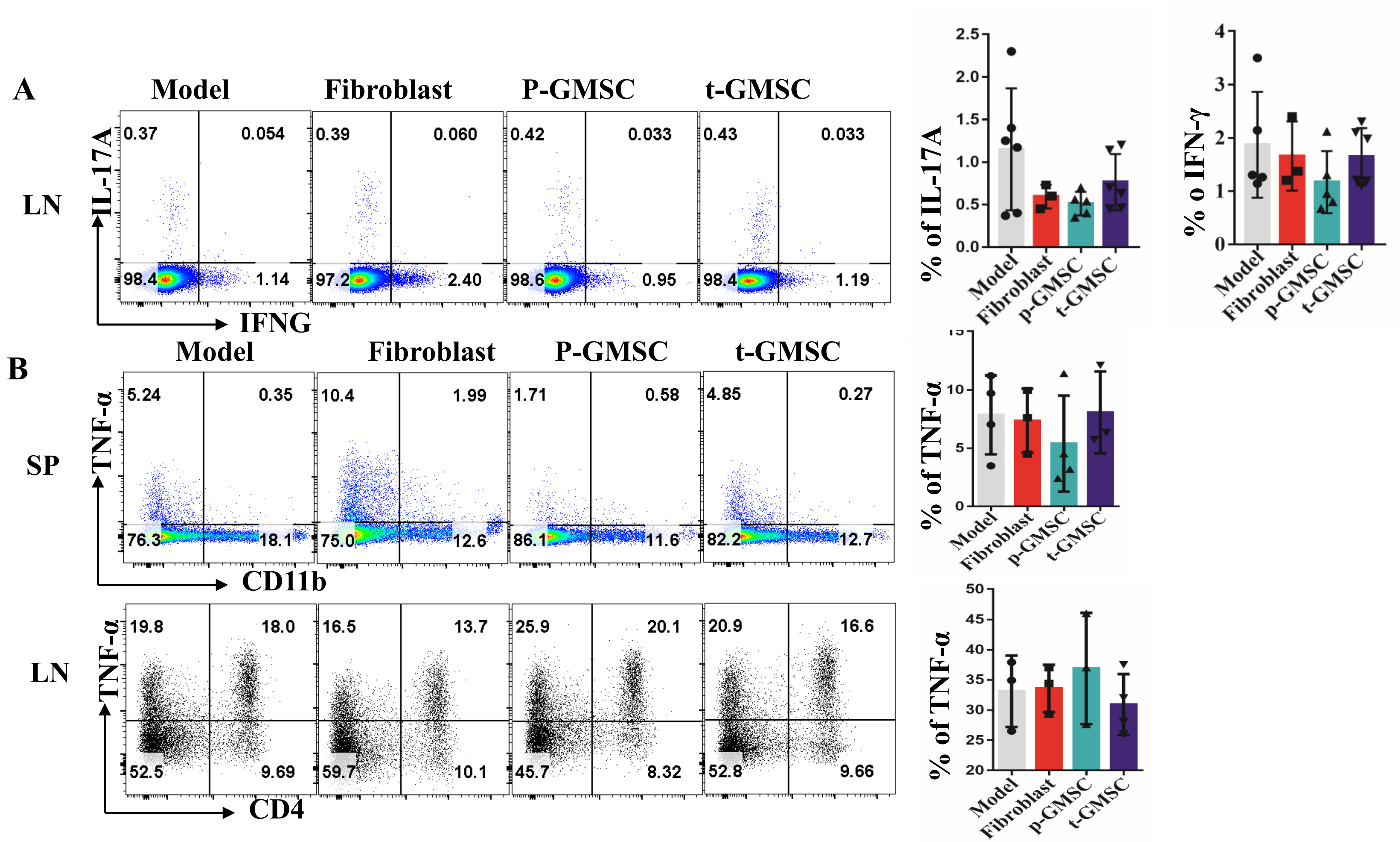

Figure S10. Fresh spleen cells and draining lymph nodes cells of ApoE$^{-/-}$mice were isolated and stimulated with phorbol12-myristate13-acetate (PMA, 0.05ug/mL) and ionomycin(0.5ug/mL) for 1h followed with brefeldin A (5ug/mL) for 4h at 37℃ in a humidified tissue culture incubator with 5% CO_2 and 95% O_2. (A): draining lymph nodes cells were stained with anti-IL-17A and IFN- γ mAbs and iving cells were gated for further analysis (Model, n=5; Fibroblast, n=3; p-GMSC, n=5; t-GMSC, n=5). (B): Fresh spleen cells were stained with anti-CD4 and TNF- α mAbs and iving cells were gated for further analysis (Model, n=4; Fibroblast, n=3; p-GMSC, n=5; t-GMSC, n=5) . (C): draining lymph nodes cells were stained with anti-CD4 and TNF- α mAbs and iving cells were gated for further analysis (Model, n=3; Fibroblast, n=3; p-GMSC, n=3; t-GMSC, n=4) . Representative dot plots of individual mice from each group were depicted. The Bar graphs show the Mean ± SD. *, p<0.05; **, p<0.01; ***, p<0.001.

Figure S11

A

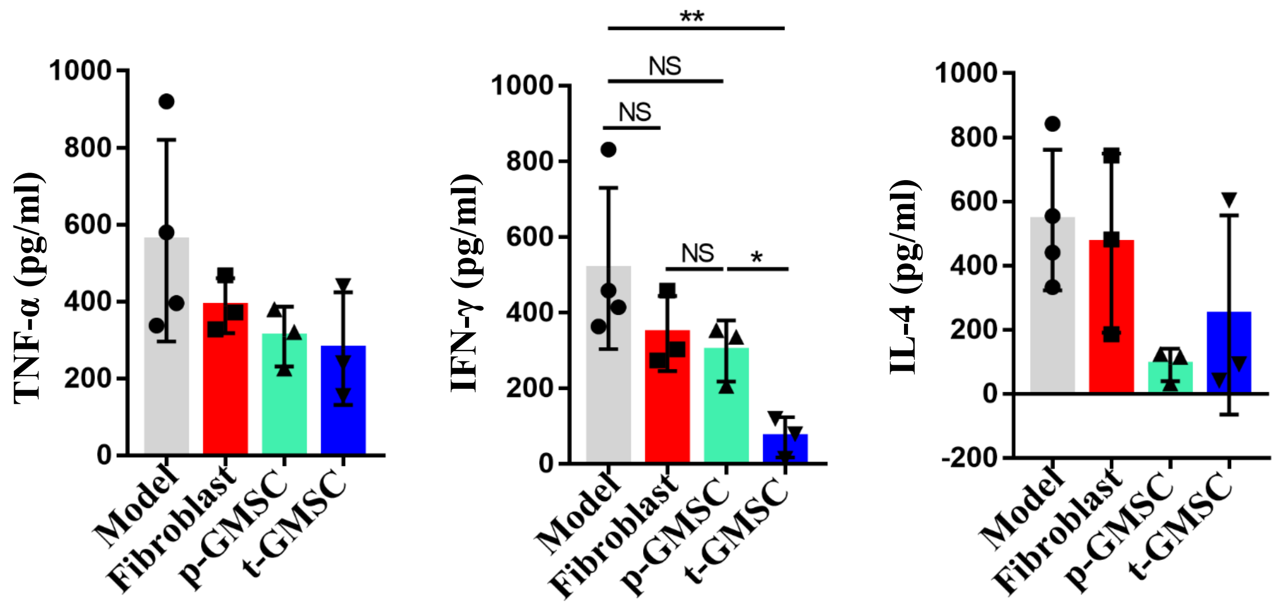

Figure S11. The effect of GMSCs on serum cytokines.
(A) Serum TNF-α, IFN-γ and IL-4 were measured by ELISA. Bar graphs show the Mean ± SD, n=3~4. *, $p<0.05$; **,
(B) $p<0.01$; ***, $p<0.001$; NS, $P\geq0.05$.

Figure S12

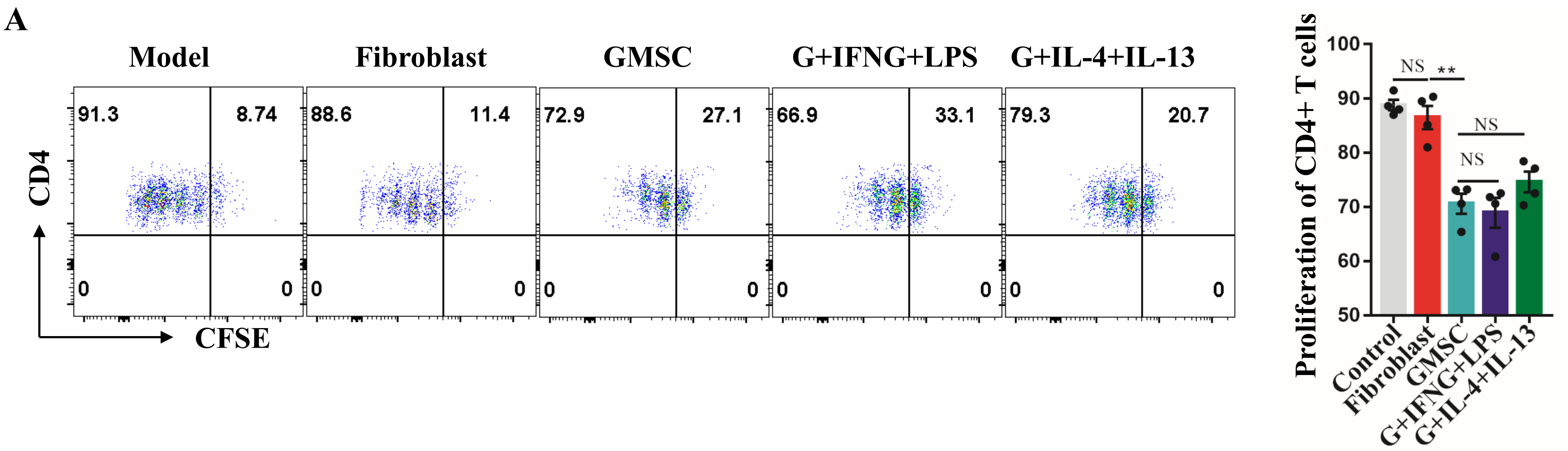

Figure S12. The effect of IFN-γ/LPS or IL-4/IL-13 on the immuno-regulatory function of GMSCs.

Mouse enriched T cells labeled with 5,6-carboxyfluorescein succinimidyl ester (CFSE) were cocultured with G-MSCs (or a human fibroblast cell line [hFibroblast] as control) in a proportion of 1:2 and stimulated with soluble anti-CD3 and mitomycin C treated non-T cells for 72 hours. Suppression of CD4+ T cell proliferation was analyzed by flow cytometry. Representative dot plots of each groups are shown(A). The Bar graphs(C) show the Mean ± SEM, n=3. *, $p<0.05$; **, $p<0.01$; ***, $p<0.001$; NS, $P\geq0.05$.
